# Lipocalin-2 drives brain metastatic progression through reciprocal tumor-microenvironment interactions in lung cancer

**DOI:** 10.1038/s41392-025-02514-2

**Published:** 2025-12-24

**Authors:** Yixiang Zhu, Jian Zhang, Danming He, Hongqing Cai, Yan He, Li Yuan, Sini Li, Yucheng Dong, Wei Zhuang, Zhijie Wang, Jianchun Duan, Xue Zhang, Zixiao Ma, Hua Bai, Jie Wang

**Affiliations:** 1 State Key Laboratory of Molecular Oncology, Department of Medical Oncology, National Cancer Center/National Clinical Research Center for Cancer/Cancer Hospital, Chinese Academy of Medical Sciences & Peking Union Medical College, Beijing, China; 2 Department of Radiation Oncology, Sichuan Clinical Research Center for Cancer, Sichuan Cancer Hospital & Institute, Sichuan Cancer Center, Affiliated Cancer Hospital of University of Electronic Science and Technology of China, Chengdu, Sichuan China; 3 Research Units of West China, Chinese Academy of Medical Sciences, West China Hospital, Chengdu, China; 4 Department of Neurosurgery, National Cancer Center/National Clinical Research Center for Cancer/Cancer Hospital, Chinese Academy of Medical Sciences and Peking Union Medical College, Beijing, China; 5 Zhejiang Cancer Hospital, Hangzhou Institute of Medicine (HIM), Chinese Academy of Sciences, Hangzhou, Zhejiang China; 6 Chinese Academy of Medical Sciences and Peking Union Medical College, Beijing, China

**Keywords:** Metastasis, Lung cancer

## Abstract

Brain metastasis is a major contributor to mortality in patients with lung cancer. The unique microenvironment of the brain plays a critical role in the initiation and progression of brain metastases (BM), yet the molecular mechanisms underlying tumor-microenvironment interactions remain poorly understood. Here, we demonstrate that upregulation of lipocalin-2 (LCN2) in tumor cells promotes brain metastatic progression by orchestrating crosstalk among metastatic tumor cells, astrocytes, and macrophages. Brain metastatic tumor cells secrete LCN2, which binds to SLC22A17 on astrocytes, activating JAK2/STAT3 signaling and inducing astrocyte activation and chemokine secretion, thereby facilitating macrophage recruitment. In turn, macrophages secrete IL-1β, which further upregulates LCN2 expression in tumor cells. Prophylactic administration of the IL-1 receptor antagonist anakinra inhibits BM formation, whereas therapeutic administration alone is ineffective. However, treatment with the STAT3 inhibitor SH4-54, either alone or in combination with anakinra, significantly suppressed tumor growth in the BM. Furthermore, tumor-secreted LCN2 can bind to SLC22A17 on tumor cells, activating JAK2/STAT3 signaling and promoting VEGF-A expression and release, which enhances tumor neovascularization. Inhibition of this axis with SH4-54, bevacizumab, or their combination effectively reduces the tumor burden in BM-bearing mice. These findings underscore the central role of LCN2 in driving brain metastasis and highlight a potential therapeutic strategy for targeting brain metastatic lung cancer.

## Introduction

The brain is a common site of metastasis in patients with lung cancer, with incidence rates reaching up to 50%.^1–5^ Despite advances in therapies targeting epidermal growth factor receptor (EGFR) and anaplastic lymphoma kinase (ALK), as well as improvements in immunotherapy and radiotherapy, the prognosis for patients with brain metastases (BM) remains dismal.^6–10^ Elucidating the molecular mechanisms underlying BM formation is therefore essential for identifying diagnostic markers and developing effective therapeutic strategies.

The brain’s microenvironment is distinct from that of extracranial sites and comprises unique cellular components, including astrocytes, microglia, oligodendrocytes, and neurons, which collectively influence the initiation and progression of BM.^11^ Astrocytes, the most abundant glial cell type in the brain, become reactive in response to stimuli. Priego et al.^12^ reported that reactive astrocytes infiltrate the periphery of metastatic lesions and secrete cytokines that disrupt tight junctions, thereby facilitating tumor invasion. Zhang et al.^13^ demonstrated that astrocyte-derived exosomes deplete PTEN in brain metastatic tumor cells, triggering cytokine chemokine (C-C motif) ligand 2 (CCL2) secretion and the recruitment of myeloid cells, ultimately promoting metastatic growth. These findings indicate that astrocytes actively remodel the brain microenvironment into a niche conducive to tumor progression. In addition to astrocytes, immune cells within the brain play critical roles in the BM. Klemm et al.^14^ compared the immune profiles of primary brain tumors and BM from lung, breast, and melanoma origins and reported that CD4⁺ T cells, monocyte-derived macrophages, and neutrophils were predominant in the BM of patients with lung cancer. Guldner et al.^15^ further showed that brain-resident myeloid cells promote an immunosuppressive niche through CXCL10 signaling, facilitating metastatic colonization. Collectively, these studies highlight the importance of the brain’s cellular microenvironment in the development of BM. However, the precise mechanisms by which tumor cells interact with astrocytes and macrophages during BM progression remain poorly defined.

Lipocalin-2 (LCN2), a member of the lipocalin protein family and an acute-phase protein, is predominantly produced by astrocytes during brain injury.^16^ In the context of BM, neutrophils also contribute to LCN2 production.^17^ LCN2 signals through several receptors, including SLC22A17 (also known as 24p3R or NGALR),^17^ LRP1,^18^ LRP2,^19^ and MC4R,^20^ with SLC22A17 garnering particular attention in cancer research.^17,21^ In models of ischemic stroke, LCN2 is markedly upregulated and enhances astrocytic phagocytic activity via LRP1, promoting neural repair. However, excessive LCN2 activity can exacerbate neuroinflammation and demyelination by inducing the production of proinflammatory cytokines.^22^ LCN2 has also been implicated in the pathogenesis of cancer brain metastasis. It has been shown to drive the inflammatory activation of astrocytes, leading to myeloid cell recruitment and facilitating metastasis in melanoma and breast cancer models.^17^ Additionally, LCN2 promotes tumor cell survival by inducing angiogenesis,^23^ enhancing epithelial-to-mesenchymal transition,^24^ and increasing iron uptake.^21^

To investigate how interactions between tumor cells and brain-resident cells contribute to BM, we performed single-cell RNA sequencing (scRNA-seq) of lung cancer BM samples and compared them to those of matched primary tumors. Our analysis identified LCN2 as a central regulator of BM progression. We found that tumor-derived LCN2 activated astrocytes through SLC22A17 and JAK2/STAT3 signaling, leading to the secretion of macrophage-recruiting chemokines. Recruited macrophages, in turn, activate IL-1β/IL-1R signaling in tumor cells, which further enhances LCN2 expression. Additionally, LCN2 binds to SLC22A17 on tumor cells themselves, activating JAK2/STAT3 and upregulating VEGF-A expression, thereby promoting tumor angiogenesis. Pharmacological inhibition of IL-1R and STAT3 signaling effectively suppressed BM growth, highlighting the therapeutic potential of targeting the LCN2 axis in lung cancer brain metastasis.

## Results

### LCN2 is overexpressed in BM and predicts poor prognosis in lung cancer patients

**Fig. 1:**
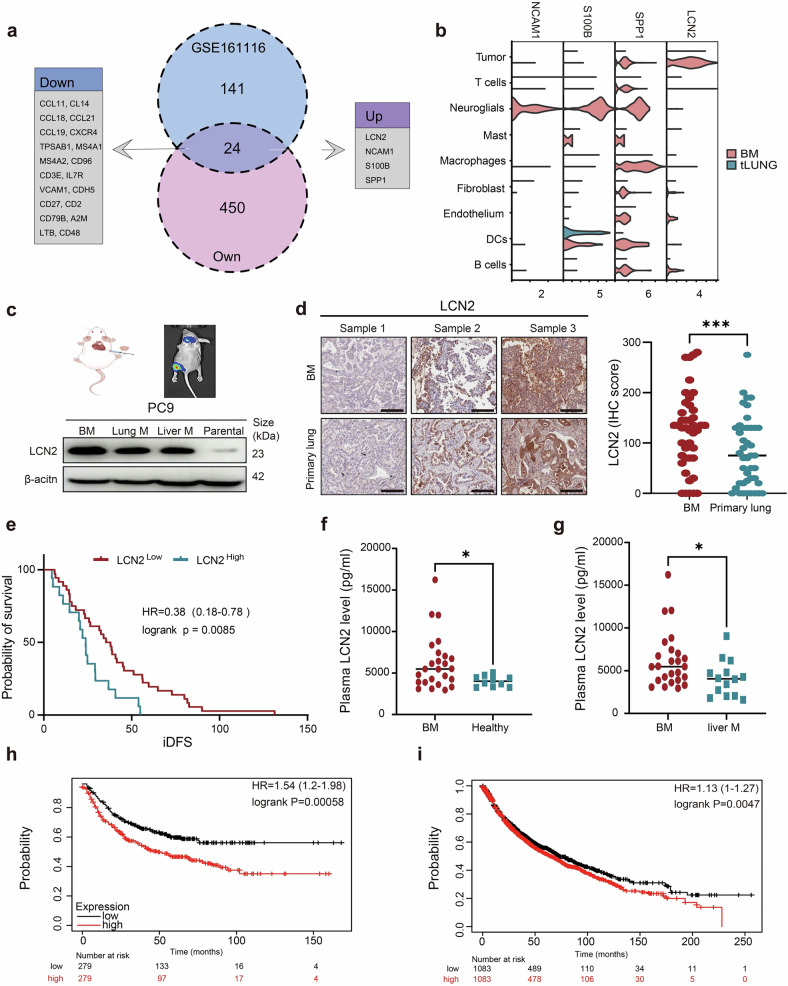
*LCN2* is overexpressed in BM and is associated with poor prognosis in lung cancer patients. **a** Venn diagram showing coupregulated and codownregulated genes in BM from lung cancer patients compared with those in primary tumors from our dataset and GSE161116. Criteria: |log₂FC| > 1, FDR < 0.05. **b** Gene expression of *LCN2*, *NCAM1*, *S100B*, and *SPP1* in tumor cells from GSE131907. **c** Schematic of the in vivo derivation of PC9-BM cells and Western blot showing LCN2 expression in metastatic variants. **d** IHC staining of 3 samples with different degrees of LCN2 expression in paired BM and primary tumors (*n* = 43). Lines represent the means and SDs; paired *t*-tests, *P < 0.05. Scale bars denote 200 μm. **e** Kaplan‒Meier analysis of iDFS based on LCN2 IHC levels in lung cancer patients with metachronous BM (*n* = 53). LCN2^high^ was defined as the upper tertile of expression levels; LCN2^low^ was defined as the upper tertile of expression levels. **f**–**g** Plasma LCN2 levels in patients with BM (*n* = 25), healthy controls (*n* = 10), and liver metastases (*n* = 14) determined via ELISA. **h**–**i** Kaplan‒Meier analysis of LCN2 mRNA levels in relation to EFS and OS in lung cancer patients (kmplot.com). BM brain metastases, IHC immunohistochemistry, ELISA enzyme-linked immunosorbent assay

To identify genes that potentially drive brain metastasis in lung cancer, we compared our own transcriptomic data^25^ with a publicly available dataset,^26^ focusing on matched primary lung tumors and corresponding brain metastatic lesions. Across both datasets, four genes—*LCN2*, *NCAM1*, *S100B*, and *SPP1*—were consistently upregulated in BM tissues compared with their matched primary tumors (Fig. 1a).

To determine the cellular specificity of these genes, we analyzed scRNA-seq data from lung cancer patients with and without BM (GSE131907).^14^ Among the four candidates, only *LCN2* was expressed predominantly in tumor cells (Fig. 1b and Supplementary Fig. 1a, b). Notably, tumor cells in the BM presented significantly higher *LCN2* expression than their counterparts in primary tumors did (Fig. 1b).

We further validated LCN2 overexpression via in vivo models. Brain metastatic (PC9-BM) cell lines were generated through intracardiac injection and recovery of metastatic lesions in mice (Fig. 1c). Western blotting revealed that LCN2 expression was markedly elevated in PC9-BM cells compared with parental PC9 cells and other metastatic variants (lung and liver).

Immunohistochemical (IHC) analysis of 43 paired patient samples revealed significantly higher LCN2 protein levels in BM than in matched primary tumors (*P* < 0.001; Fig. 1d). Clinically, high LCN2 expression in BM tissues was correlated with significantly shorter intracranial disease-free survival (iDFS) (Fig. 1e).

To assess systemic *LCN2* expression, we performed an enzyme-linked immunosorbent assay (ELISA) on plasma samples. Compared with healthy controls, BM patients presented significantly elevated plasma LCN2 levels (*P* < 0.05; Fig. 1f) and liver metastasis patients (*P* < 0.05; Fig. 1g). Moreover, Kaplan‒Meier analyses of public datasets revealed that high *LCN2* mRNA levels predict worse event-free survival (EFS) (Fig. 1h) and overall survival (OS) in lung cancer patients (Fig. 1i). Collectively, these findings establish LCN2 as a key molecule that is overexpressed in BM and is associated with poor clinical outcomes in patients with lung cancer.

### LCN2 is required for BM

**Fig. 2:**
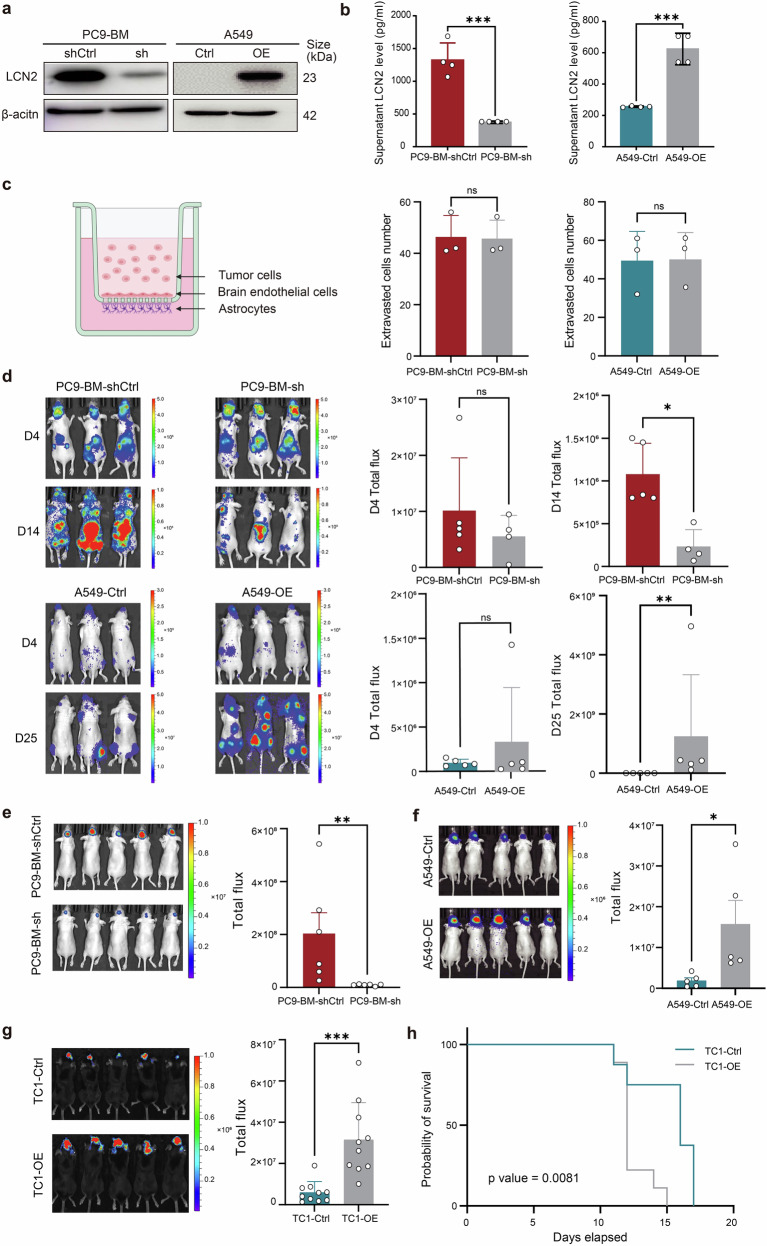
LCN2 promotes the growth of the BM but does not affect BBB transmigration. **a**, **b** Western blot and ELISA analysis of LCN2 expression in stably engineered cell lines. The data are presented as the means ± SDs, *n* = 3; two-sided *t* test, ***P < 0.001. **c** In vitro BBB transmigration assay comparing LCN2-KD PC9-BM and LCN2-OE A549 cells (*n* = 3). The data are presented as the means ± SDs, *n* = 3; two-sided *t* test, ns, not significant. **d** BLI at 4, 14, and 25 days after intracardiac injection of LCN2 KD (*n* = 4) and control PC9-BM (*n* = 5) cells and LCN2 OE (*n* = 5) and control A549 cells (*n* = 5). The data are presented as the means ± SDs; 2 replicate experiments involving LCN2 KD and control PC9-BM cells; two-sided Mann‒Whitney test, *P < 0.05, **P < 0.01. **e**–**g** BLI at 8, 12, and 8 days after intracranial injection of LCN2-KD (*n* = 5) and control PC9-BM (*n* = 5) cells, LCN2-OE (*n* = 5) and control A549 (*n* = 5) cells, LCN2-OE (*n* = 10) and control TC1 (*n* = 10) cells. The data are presented as the means ± SDs; two-sided Mann‒Whitney test, *P < 0.05, **P < 0.01, ***P < 0.001. **h** Kaplan‒Meier curve showing that the BM OS of mice was evaluated in LCN2 OE (*n* = 10) and control TC1 cells (*n* = 10). KD knockdown, OE overexpression, BBB blood‒brain barrier, BM brain metastases, OS overall survival

To delineate the functional role of LCN2 in lung cancer brain metastasis, we first evaluated its expression across multiple lung cancer cell lines (A549, PC9, H358, H23, KLN205, LLC, and TC1) (Supplementary Fig. 2a). We then engineered several cell lines—A549, TC1 and PC9-BM—to stably overexpress or knock down LCN2 (Fig. 2a, b and Supplementary Fig. 5e).

To assess whether LCN2 regulates the ability of tumor cells to infiltrate the brain parenchyma, we conducted migration and transmigration assays. LCN2 knockdown in PC9-BM cells and LCN2 overexpression in A549 cells did not significantly affect cell migration (Supplementary Fig. 2b–c). Additionally, LCN2 manipulation did not alter tumor cell transmigration across the blood‒brain barrier (BBB) in an in vitro BBB model, nor did it disrupt brain endothelial cell adhesion (Fig. 2c; Supplementary Fig. 2d).

Consistent with the in vitro findings, in vivo BM models generated via intracardiac injection of PC9-BM or A549 cells revealed that LCN2 had no effect on the transmigration of tumor cells across the BBB (Fig. 2d). Similarly, carotid artery injection experiments revealed comparable BBB penetration in both control LCN2-manipulated groups (Supplementary Fig. 3a). These data indicate that LCN2 does not impact the ability of tumor cells to cross the BBB.

Strikingly, once tumor cells reach the brain parenchyma, LCN2 expression significantly enhances intracranial tumor growth (*P* < 0.05 and *P* < 0.01, respectively; Fig. 2d). Intracranial injection of PC9-BM cells with LCN2 knockdown resulted in markedly reduced the brain tumor burden compared to controls (*P* < 0.01; Fig. 2e). Conversely, A549 (Fig. 2f) and TC1 (Fig. 2g) cells overexpressing *LCN2* formed significantly larger tumors in the brain than their control counterparts did (*P* < 0.05 and *P* < 0.001, respectively). Notably, *LCN2* overexpression also led to reduced OS in BM-bearing mice (Fig. 2h).

In summary, LCN2 is dispensable for BBB transmigration but is essential for promoting tumor growth within the brain microenvironment. These results highlight LCN2 as a critical driver of metastatic colonization and progression in lung cancer BM.

### LCN2 promotes angiogenesis of lung cancer BM

**Fig. 3:**
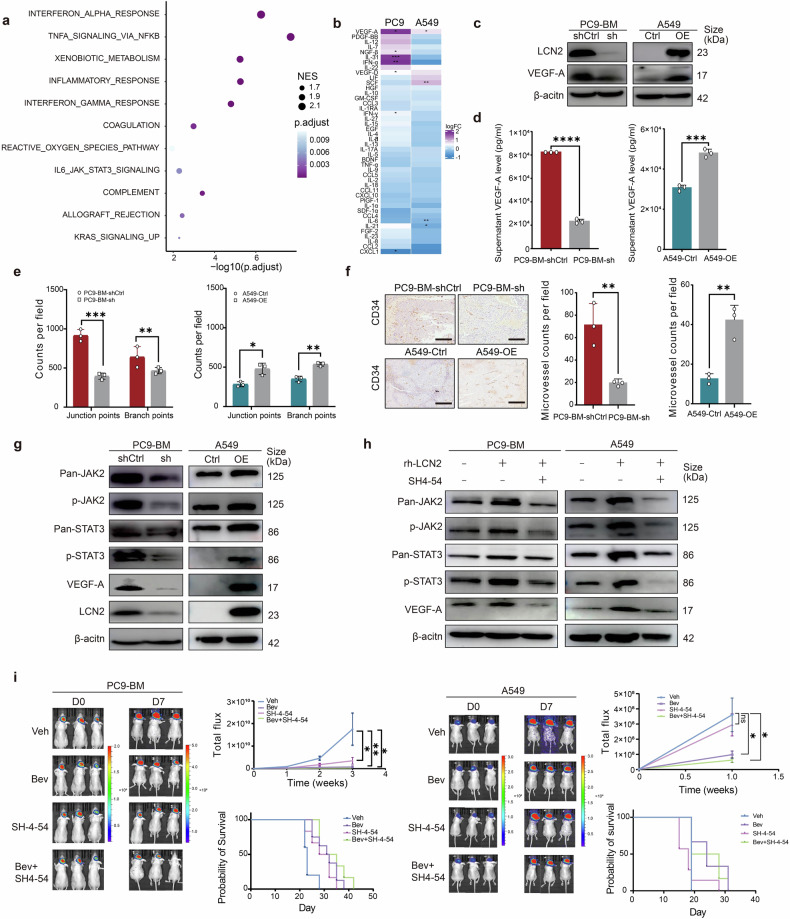
LCN2 promotes angiogenesis in BM from lung cancer patients. **a** Pathway enrichment analysis of lung cancer tumor cells with high versus low LCN2 expression. **b** Heatmap of cytokine antibody arrays in PC9-BM control and PC9-BM KD cells, as well as A549 control and A549 OE cells. Each group included three biological replicates. The data are presented as log fold changes and were normalized to the corresponding cell line with lower LCN2 expression. *FDR < 0.05, **FDR < 0.01; *P* values were adjusted via the Benjamini‒Hochberg method. **c** Western blot analysis of LCN2 expression in PC9-BM control, PC9-BM KD, A549 control, and A549 OE cells. **d** ELISA quantification of LCN2 levels in PC9-BM control, PC9-BM KD, A549 control, and A549 OE cells (*n* = 3). The data are presented as the means ± SDs; two-sided *t* test, ****P < 0.0001. **e** Quantification of junction and branch points per field (*n* = 3) of angiogenesis in PC9-BM control, PC9-BM KD, A549 control, and A549 OE cells. The data are presented as the means ± SDs; two-sided *t* test, * *P* < 0.05, ** *P* < 0.01, ***P < 0.001. **f** Representative immunohistochemistry (IHC) images of CD34 expression. The quantified data are shown as the means ± SDs (*n* = 3); two-sided *t*-test, **P < 0.01. Scale bars, 200 μm. **g** Western blot analysis of phosphorylated JAK2 (p-JAK2), phosphorylated STAT3 (p-STAT3), and VEGF-A. **h** Western blot analysis of the indicated cells treated with or without 100 ng/mL recombinant human LCN2 (rhLCN2), with or without the STAT3 inhibitor SH4-54 (1 μM). **i** The tumor inhibition rate and survival analysis were performed by normalizing the tumor burden to that of vehicle-treated BM-bearing mice. LCN2 lipocalin-2, BM brain metastases, IHC immunohistochemistry

To elucidate the role of LCN2 in BM, we conducted Gene Ontology (GO) analysis using scRNA-seq data (Supplementary Fig. 4). The analysis revealed activation of the inflammatory response and the IL6–JAK–STAT3 signaling pathways in tumor cells with high LCN2 expression (Fig. 3a). To identify downstream effectors, we profiled cytokine expression in the supernatants of LCN2-knockdown (KD) PC9-BM cells, LCN2-overexpressing (OE) A549 cells, and their respective controls via a Luminex multiplex assay. Compared with those in control cells, VEGF-A levels were significantly lower in LCN2-KD PC9-BM cells and elevated in LCN2-OE A549 cells (Fig. 3b). These findings were corroborated by western blotting (Fig. 3c) and ELISA (Fig. 3d), which confirmed increased VEGF-A expression and secretion in LCN2-high cells relative to their LCN2-low counterparts in both PC9-BM (*P* < 0.0001) and A549 (*P* < 0.001) cells.

Given the established role of VEGF-A in angiogenesis, we next examined whether LCN2 promotes angiogenesis in the BM. Conditioned medium from LCN2-high cells significantly enhanced brain endothelial tube formation compared to medium from LCN2-low cells in both the PC9-BM (*P* < 0.001 and *P* < 0.01) and A549 (*P* < 0.05 and *P* < 0.01) cell lines (Fig. 3e; Supplementary Fig. 3b). Recombinant VEGF-A further promoted endothelial tube formation, an effect that was abolished by the anti-VEGF antibody bevacizumab (Supplementary Fig. 3c). Consistently, in vivo IHC analysis revealed significantly greater vessel density in BM formed by LCN2-high cells than in BM formed by LCN2-low cells (*P* < 0.01) (Fig. 3f). Collectively, these findings indicate that LCN2 facilitates robust angiogenesis in BM tumors.

GO analysis also revealed activation of the tumor necrosis factor (TNF) and Janus kinase (JAK)-STAT signaling pathways in PC9-BM cells (Supplementary Fig. 5a) and in tumor cells with high LCN2 expression in clinical BM patient samples (Fig. 3a). Correspondingly, western blotting revealed that LCN2-KD decreased the phosphorylation of JAK2 and STAT3 in PC9-BM cells, whereas LCN2-OE increased their phosphorylation in A549 cells (Fig. 3g). No significant changes were observed in the phosphorylation of NF-κB, JAK1, or JAK3 (Supplementary Fig. 5b–g). Moreover, recombinant LCN2 (rhLCN2) increased both JAK2/STAT3 phosphorylation and VEGF-A expression in PC9-BM and A549 cells, effects that were abolished by the STAT3 inhibitor SH4-54 (Fig. 3h). These results suggest that LCN2 induces VEGF-A upregulation through the JAK2/STAT3 signaling axis.

Therapeutically, treatment of PC9-BM LCN2-high BM-bearing mice with SH4-54, bevacizumab, or a combination of both significantly suppressed tumor progression and prolonged OS. In contrast, only bevacizumab was effective in the A549 LCN2-low model (Fig. 3i). These data reveal that the LCN2–JAK2–STAT3 axis is a crucial regulator of VEGF-A-driven angiogenesis, suggesting that the LCN2–JAK2–STAT3 axis is a promising therapeutic target for lung cancer patients with BM characterized by high LCN2 expression.

### LCN2 mediates astrocyte activation and macrophage recruitment in lung cancer BM

To investigate whether LCN2 specifically contributes to lung cancer BM, we established both subcutaneous and orthotopic pulmonary tumor models. LCN2 expression did not influence tumor growth in either model (Supplementary Fig. 6a, b). Similarly, in subcutaneous tumors, blood vessel density was comparable between tumors derived from LCN2-high- and LCN2-low-expressing cells (Supplementary Fig. 6c). These findings suggest that LCN2-dependent tumor promotion may be specific to the brain microenvironment.

**Fig. 4:**
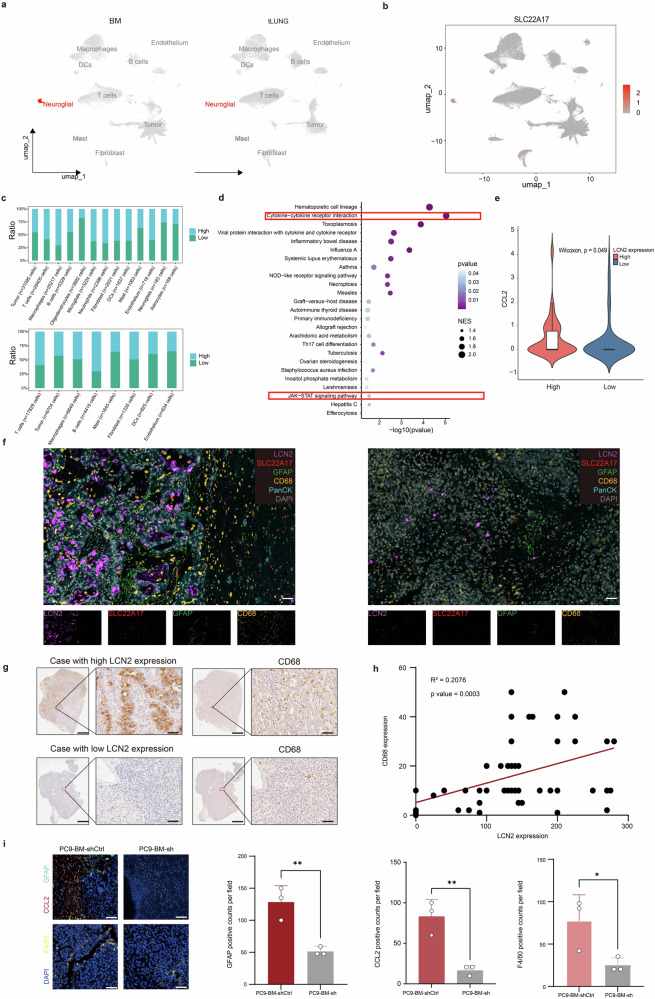
LCN2 enhances cancer cell–astrocyte interactions. **a** Comparison of microenvironmental cell populations between BM and primary tumors in lung cancer patients via scRNA-seq. **b** Expression profile of SLC22A17 (LCN2 receptor) across cell subtypes. **c** Distribution of microenvironmental cells in BM and primary tumors stratified by tumor cell LCN2 expression. In the BM cohort (above), samples with fewer than 10 tumor cells were initially filtered out. Subsequently, both the BM and primary tumor cohorts were categorized into LCN2-high and LCN2-low groups based on the mean normalized LCN2 expression level (BM: *n* = 12 per group; Primary tumor: *n* = 5 per group). **d** Pathway enrichment analysis of astrocytes associated with high versus low *LCN2* expression in tumor cells. **e** Expression of *CCL2* in astrocytes in the context of high versus low *LCN2* expression in tumor cells. **f** Multiplex IHC images showing heterogeneity in the tumor immune microenvironment (TIME) of lung cancer with BM samples with high or low LCN2 expression; samples were stained for LCN2, SLC22A17, GFAP, CD68, panCK, and DAPI. Scale bar, 50 μm. **g** Representative IHC images of CD68 expression in LCN2-high and LCN2-low BM samples from lung cancer patients. Scale bars, 2000 μm and 200 μm. **h** Pearson correlation analysis of LCN2 and CD68 protein levels in NSCLC BM samples. **i** Representative immunofluorescence images of GFAP, CCL2, F4/80, and DAPI staining in PC9-BM-shCtrl and PC9-BM-sh cells. The data are presented as the means ± SDs, *n* = 3; two-sided *t*-test, * *P* < 0.05, **P < 0.01

We next compared the tumor microenvironments of BM and primary lung tumors from lung cancer patients via scRNA-seq (Supplementary Fig. 1). Neuroglial cells—specifically astrocytes and oligodendrocytes—were exclusive to the brain (Fig. 4a) and selectively expressed high levels of *SLC22A17*, the receptor for LCN2 (Fig. 4b). Notably, SLC22A17 is expressed predominantly in brain tissue under physiological conditions (Supplementary Fig. 7). A previous study demonstrated that LCN2 derived from granulocytes activates astrocytes, triggering the recruitment of myeloid cells to the brain.^17^ Our scRNA-seq data revealed that BM with high LCN2 expression presented an increased proportion of infiltrating macrophages, whereas no such difference was observed in primary tumor tissues (Fig. 4c).

In the LCN2-high group, GO analysis of astrocytes revealed enrichment in cytokine–cytokine receptor interaction pathways (Fig. 4d), including elevated expression of *CCL2*, a key chemokine for macrophage recruitment (Fig. 4e). Similar transcriptional changes were observed in oligodendrocytes (Supplementary Fig. 8a, b). Multiplex IHC staining of BM tissues from lung cancer patients confirmed the presence of activated astrocytes and macrophages in tumors with high *LCN2* expression (Fig. 4f).

We further validated these observations in a cohort of 58 BM tissue samples via IHC (Fig. 4g). LCN2 protein levels were positively correlated with those of CD68, a macrophage marker (Fig. 4h). In vivo, astrocyte and macrophage activation was significantly reduced in BM-bearing mice implanted with LCN2-knockdown cells (*P* < 0.01, *P* < 0.01, and *P* < 0.05; Fig. 4i), whereas no changes were observed in subcutaneous tumors (Supplementary Fig. 8c). These findings suggest that high LCN2 expression in tumor cells remodels the brain TME by promoting astrocyte activation and macrophage infiltration.

To further examine whether astrocytes mediate LCN2-driven macrophage recruitment, we assessed macrophage migration via conditioned media from cocultures of tumor cells and astrocytes. Conditioned medium from LCN2-high tumor cells cocultured with astrocytes significantly enhanced macrophage migration (Supplementary Fig. 8d), whereas conditioned media from LCN2-overexpressing tumor cells alone (Supplementary Fig. 8e) or cocultured with oligodendrocytes (Supplementary Fig. 8f) had no such effect. Consistently, elevated *CCL2* levels were detected in supernatants from LCN2-high tumor cells cocultured with astrocytes (Supplementary Fig. 8g) but not in supernatants from tumor cells alone (Supplementary Fig. 8i) or from those cocultured with oligodendrocytes (Supplementary Fig. 8j).

Collectively, these results indicate that LCN2 facilitates cross-talk between tumor cells and astrocytes in the brain, activating astrocytes to secrete CCL2, thereby promoting macrophage recruitment and contributing to a protumorigenic microenvironment in the BM of lung cancer patients.

### LCN2 activates astrocytes via JAK2/STAT3 signaling

**Fig. 5:**
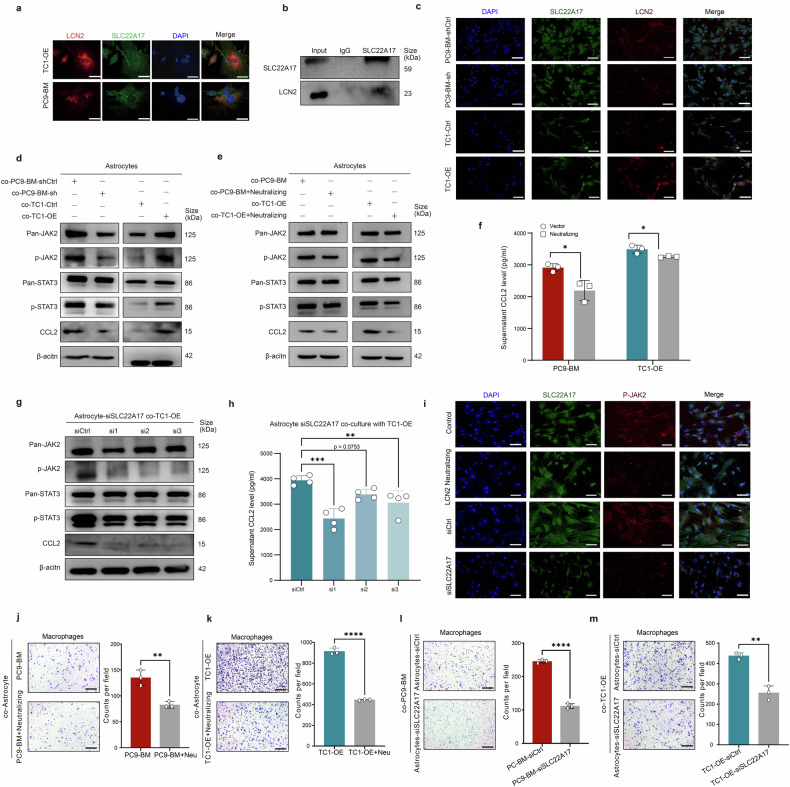
LCN2 activates astrocytes by engaging in JAK2/STAT3 signaling. **a** Representative immunofluorescence images of LCN2, SLC22A17, and DAPI in TC1-OE and PC9-BM cells. Scale bar, 20 μm. **b** Immunoprecipitation showing the interaction between LCN2 and SLC22A17 in astrocytes cocultured with TC1-OE cells. **c** Representative IF images showing LCN2–SLC22A17 binding in PC9-BM-shCtrl, PC9-BM-sh, TC1-Ctrl, and TC1-OE cells. Scale bar, 100 μm. **d**, **e** Western blots showing phosphorylated JAK2/STAT3 and CCL2. **f** ELISA quantification of CCL2 in astrocytes cocultured with PC9-BM control, PC9-BM KD, TC1 control, or TC1-OE cells treated with 100 ng/mL LCN2-neutralizing antibody for 18 h (*n* = 3). The data are presented as the means ± SDs; two-sided *t* test, *P < 0.05. **g** Western blot showing p-JAK2, p-STAT3, and CCL2 levels in astrocytes transfected with *SLC22A17* siRNAs for 48 hours and cocultured with TC1-OE cells. **h** ELISA showing CCL2 levels in astrocytes transfected with *SLC22A17* siRNAs and cocultured with TC1-OE cells. **i** IF staining of astrocytes cocultured with PC9-BM cells ± LCN2-neutralizing antibody (100 ng/mL) showing the subcellular localization of SLC22A17 and p-JAK2 after 18 hours. Scale bar, 100 μm. **j**, **k** Representative images and quantification of macrophage recruitment in astrocytes cocultured with PC9-BM or TC1-OE cells ± LCN2-neutralizing antibody. The data are presented as the means ± SDs, *n* = 3; two-sided *t*-test, **P < 0.01, ****P < 0.0001. Scale bar, 200 μm. **l**–**m** Representative images and quantification of macrophages in astrocytes transfected with SLC22A17 siRNAs and cocultured with PC9-BM or TC1-OE cells ± LCN2-neutralizing antibody. The data are presented as the means ± SDs, *n* = 3; two-sided *t*-test, **P < 0.01, ****P < 0.0001. Scale bar, 200 μm

To elucidate the molecular mechanisms by which LCN2 activates astrocytes, we conducted IF and immunoprecipitation (IP) assays. IF staining demonstrated the colocalization of LCN2 with SLC22A17 in astrocytes (Fig. 5a). IP confirmed the physical interaction between LCN2 and SLC22A17 in astrocytes cocultured with TC1-OE cells (Fig. 5b). Coculturing astrocytes with LCN2-knockdown (KD) PC9-BM cells reduced this interaction, while *LCN2* OE in TC1 cells increased this interaction (Fig. 5c). These results suggest that LCN2–SLC22A17 binding is essential for the prometastatic activity of LCN2 in the brain.

To identify downstream signaling pathways, GO analysis of astrocytes in the scRNA-seq dataset revealed JAK2–STAT3 pathway activation in the context of high LCN2 expression in tumor cells (Fig. 4d). A previous study reported a direct interaction between SLC22A17 and JAK2 in pancreatic cancer cells.^27^ We found that astrocytes cocultured with LCN2-high tumor cells presented increased phosphorylation of JAK2 and STAT3, whereas no significant changes in JAK1, JAK3, or STAT1 levels were detected (Supplementary Fig. 9a, b).

Using the JASPAR database (https://jaspar.elixir.no/analysis), we identified potential STAT3 binding motifs in the *CCL2* promoter, suggesting transcriptional regulation by STAT3 (Supplementary Fig. 9c). ELISA confirmed that STAT3 inhibition significantly decreased CCL2 secretion in astrocytes cocultured with PC9-BM or A549-OE cells (Supplementary Fig. 9d). Furthermore, treatment with 100 ng/mL LCN2-neutralizing antibody for 18 h significantly reduced the levels of phosphorylated JAK2, STAT3, and CCL2 in astrocytes cocultured with PC9-BM (*P* < 0.05) and TC1-OE (*P* < 0.05) cells (Fig. 5d–f). Similarly, *SLC22A17* knockdown markedly suppressed LCN2-induced phosphorylation of JAK2/STAT3 and CCL2 expression (Fig. 5g-i).

Importantly, both LCN2 neutralization and SLC22A17 inhibition significantly reduced macrophage recruitment (Fig. 5j–m). These findings indicate that LCN2 activates astrocytes by binding to SLC22A17 and triggers JAK2–STAT3 signaling, leading to the upregulation of *CCL2* and the recruitment of macrophages, thereby remodeling the brain metastatic microenvironment.

### Macrophages promote LCN2 expression in brain metastatic tumor cells via the IL-1β–IL-1R–NF-κB signaling axis

**Fig. 6:**
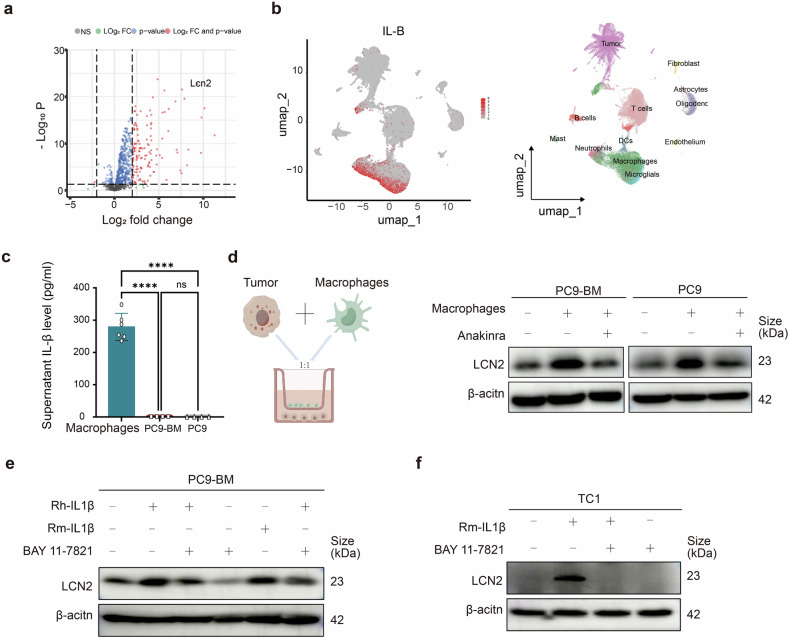
Macrophages activate the IL-1β–IL-1R–NF-κB pathway to promote tumor-derived LCN2 expression. **a** *LCN2* mRNA levels were significantly increased after 24 hours of IL-1β treatment in pancreatic cancer cells. **b** Expression profile of *IL-1B* across different cell types in brain and primary lung tumors (scRNA-seq). **c** ELISA quantification of IL-1β levels in macrophages (*n* = 6), PC9-BM (*n* = 4), and PC9 cells (*n* = 4). The data are presented as the means ± SDs; two-sided *t* test, ****P < 0.0001; ns, not significant. **d** Western blot analysis of PC9 and PC9-BM cells cocultured with macrophages with or without anakinra (an IL-1R inhibitor). **e**, **f** Western blot analysis of LCN2 expression in PC9 and PC9-BM cells treated with recombinant IL-1β ± BAY 11-7082 (NF-κB inhibitor)

To elucidate the mechanism underlying elevated LCN2 expression in BM, GO analysis revealed enrichment of the TNF–NF-κB signaling pathway in tumor cells with high LCN2 expression (Fig. 3a). Given that FOS and JUN—downstream targets of IL-1β—are regulated by NF-κB, we hypothesized that IL-1β might drive *LCN2* expression. Public database analysis confirmed a significant increase in *LCN2* mRNA levels in pancreatic cancer cells treated with IL-1β for 24 h compared with those in untreated controls (Fig. 6a).

ScRNA-seq of lung cancer BM samples revealed that *IL-1B* was predominantly expressed by macrophages, microglia, and neutrophils (Fig. 6b). Consistent with these findings, ELISA revealed significantly greater IL-1β secretion by macrophages than by either BM tumor cells or parental PC9 cells (Fig. 6c). Furthermore, coculture of macrophages with tumor cells increased LCN2 protein levels, an effect that was abolished by the IL-1R antagonist anakinra, indicating that IL-1β–IL-1R signaling mediates macrophage-induced LCN2 upregulation (Fig. 6d).

Treatment with recombinant IL-1β significantly increased LCN2 protein expression in both PC9-BM and TC1 cells. This induction was blocked by the NF-κB inhibitor BAY 11-7082, implicating NF-κB as a downstream effector (Fig. 6e, f). These results collectively demonstrate that macrophages enhance LCN2 expression in BM tumor cells through the IL-1β–IL-1R–NF-κB signaling pathway.

### Targeting IL-1β–IL-1R–mediated LCN2 induction suppresses BM

**Fig. 7:**
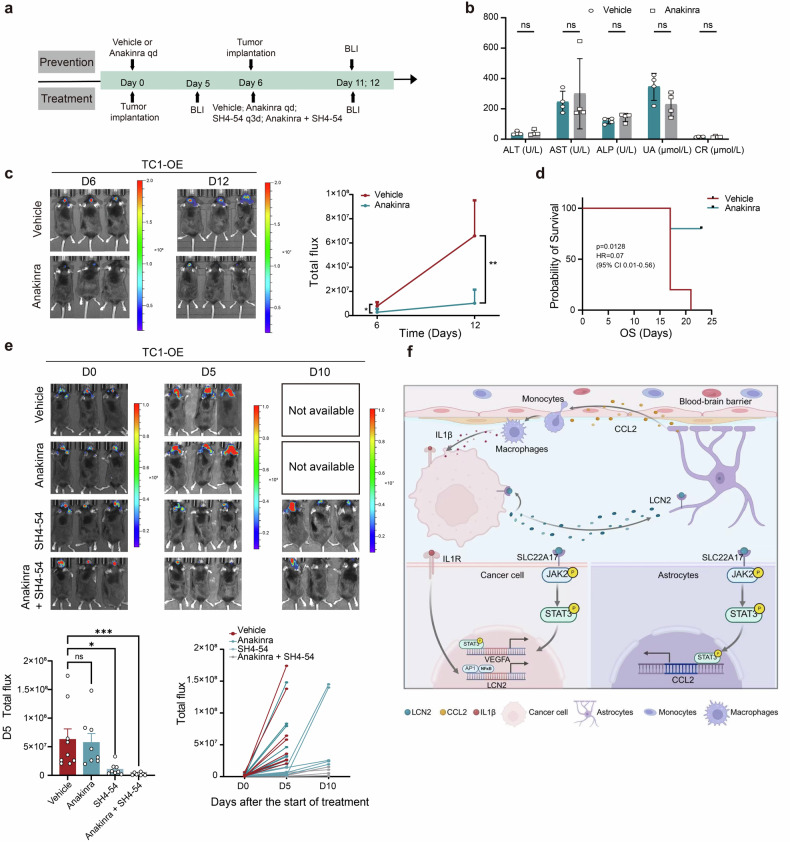
Preventive inhibition of IL-1β–IL-1R signaling reversed LCN2 expression to inhibit BM. **a** Schematic outlining prevention and treatment protocols in the BM mouse model. **b** Safety profile of the Anakinra- and vehicle-treated groups; the data are presented as the means ± SDs, *n* = 4; two-sided *t*-test, ns not significant. **c**, **d** Tumor inhibition rate and survival analysis in the prevention cohort, normalized to vehicle-treated BM-bearing mice (*n* = 5). **e** Bioluminescence imaging (BLI) on days 0, 5, and 10 posttreatment in BM-bearing mice. The treatment groups included vehicle, anakinra (100 mg/kg daily), SH4-54 (10 mg/kg every 3 days), and anakinra + SH4-54. The data are presented as the means ± SDs, *n* = 9; two-way ANOVA, ns not significant, * *P* < 0.05, ***P < 0.001. **f** Summary diagram of the proposed mechanism (created with BioRender.com). LCN2 lipocalin-2, BM brain metastases, IHC immunohistochemistry

To evaluate whether inhibition of the IL-1β–IL-1R axis could prevent or treat lung cancer brain metastasis, we performed both prevention and treatment studies in mouse models (Fig. 7a). Compared with vehicle treatment, anakinra administration did not induce liver or kidney toxicity (Fig. 7b).

In the prevention arm, anakinra significantly inhibited BM tumor growth (*P* < 0.01) (Fig. 7c) and prolonged OS (Fig. 7d). However, anakinra treatment after BM establishment failed to suppress tumor growth. In contrast, the STAT3 inhibitor SH4-54 alone, which was more effective in combination with anakinra, significantly inhibited tumor progression (Fig. 7e).

IHC analysis revealed reduced LCN2 expression (Supplementary Fig. 10a) and decreased macrophage infiltration (Supplementary Fig. 10b, c) in the anakinra prevention group compared with the vehicle group. These findings suggest that early inhibition of IL-1β–IL-1R signaling can suppress LCN2-driven tumor progression and that combining anakinra with STAT3 inhibition is a promising therapeutic strategy for lung cancer brain metastasis.

## Discussion

The formation of the BM is a highly selective and complex process shaped by the unique microenvironment of the brain. In this study, we identified LCN2 as a pivotal factor predominantly expressed in lung cancer BM tumor cells, where it plays a crucial role in facilitating metastatic colonization. We demonstrated that tumor-derived LCN2 activates astrocytes within metastatic foci, triggering macrophage recruitment. These macrophages, in turn, secrete IL-1β, which upregulates LCN2 expression in tumor cells, establishing a paracrine feedback loop. Prophylactic inhibition of the IL-1β–IL-1R axis significantly suppressed BM progression in vivo. Additionally, LCN2 binds to its receptor SLC22A17 on tumor cells, activating the JAK2–STAT3 signaling pathway, which elevates VEGF-A expression and secretion, thereby promoting tumor neovascularization. Pharmacologic inhibition of STAT3 or VEGF-A suppressed tumor growth in BM-bearing mice (Fig. 7i).

Crosstalk between tumor cells and the brain-specific TME is critical for metastatic colonization and outgrowth. Astrocytes, the most abundant glial cells in the brain, play dual roles depending on the stage of metastasis, exerting both antimetastatic effects and prometastatic effects.^28^ In agreement with Alder’s findings,^17^ our study revealed that LCN2-activated astrocytes secrete inflammatory mediators that contribute to an immunosuppressive milieu. Notably, using both in vitro assays and in vivo models of carotid artery and left ventricular injection, we found that LCN2-induced astrocyte activation does not compromise BBB integrity in BM from patients with lung cancer. This contrasts with nonmalignant neurological diseases, where high LCN2 levels can disrupt the BBB through astrocyte pyroptosis^16^ and other mechanisms,^29^ suggesting that the functional phenotype of LCN2-activated astrocytes is context dependent. Our study further underscores a distinct BM mechanism in lung cancer. Unlike in breast cancer and melanoma, where stromal cells—particularly granulocytes—are the main source of LCN2,^17^ we found that LCN2 is predominantly expressed by tumor cells in the BM of lung cancer patients. Although granulocytes in the lung cancer BM TME may also contribute to LCN2, tumor-specific knockdown of LCN2 markedly reduces macrophage infiltration and the metastatic burden. These data support the hypothesis that tumor-derived LCN2 initiates the prometastatic feedback loop in the lung cancer BM model, whereas TME-derived LCN2 may amplify, but not initiate, this process. Therefore, the role of LCN2 in astrocyte-mediated inflammation and BM progression likely arises from its production by multiple cellular sources.

Brain endothelial cells represent a key component of the TME and are instrumental in metastatic initiation and growth. VEGF-A facilitates interactions between cancer cells and the brain endothelium. We observed that VEGF-A expression and secretion were elevated in tumor cells with high LCN2 expression, which was correlated with increased microvascular density in BM model mice. Notably, STAT3 activation—known to upregulate VEGF, MMP2, and PLK1—was significantly increased in LCN2-overexpressing tumor cells.^30–32^ While a previous study by Huang et al.^27^ showed that LCN2 binding to SLC22A17 activates JAK2–STAT3 signaling to drive CXCL1 expression, our findings demonstrate that in brain tumor cells expressing SLC22A17, LCN2 binding similarly activates JAK2–STAT3 but leads instead to VEGF-A upregulation. Importantly, inhibition of STAT3 (with SH4-54) and VEGF-A (with bevacizumab) partially reversed LCN2-driven tumor growth in BM-bearing mice. However, LCN2 did not impact tumor growth or angiogenesis in subcutaneous or orthotopic lung tumor models, highlighting its specific role within the brain metastatic niche. In our lung cancer BM model, STAT3 inhibition—either alone or in combination with anti-VEGF-A monoclonal antibodies—robustly impeded BM progression. In addition to suppressing VEGF-A expression, STAT3 inhibitors also exhibit immunomodulatory properties. Preclinical and early-phase clinical studies have shown that STAT3 blockade can reverse the immunosuppressive phenotype of pSTAT3-positive astrocytes in the BM.^12^ Currently, two phase II trials are underway: one evaluating STAT3 inhibitors as adjuvant therapy postresection of BM (NCT05689619) and another comparing whole-brain radiation therapy (WBRT) alone with WBRT plus STAT3 inhibition in patients with multiple BM or leptomeningeal spread (NCT05793489). Nonetheless, not all patients respond to STAT3 inhibition, and predictive biomarkers for therapeutic response remain elusive. In our study, STAT3 inhibition was effective in the PC9-BM model but not in the A549 model, suggesting that LCN2 expression may serve as a predictive biomarker for STAT3-targeted therapy in lung cancer BM. Thus, our findings broaden the mechanistic and therapeutic rationale for applying STAT3 inhibition in the BM setting.

We also delineated the functional significance of the IL-1β–LCN2 axis in lung cancer BM. While theoretical models suggest that IL-1β blockade should downregulate LCN2 and suppress BM, we found that anakinra (an IL-1R antagonist) was only effective when it was administered prophylactically. Among currently available IL-1 inhibitors (e.g., canakinumab, rilonacept, and gevokizumab) and the IL-1R inhibitor anakinra, the latter has the highest—but still limited—BBB permeability.^33^ Drug delivery timing may be critical; prophylactic treatment likely allows for greater drug penetration into premetastatic lesions, whereas therapeutic administration in established metastases is less effective. Additionally, IL-1β-independent pathways, such as TNF-α signaling, may sustain LCN2 expression in advanced lesions, thereby reducing reliance on IL-1β. As tumor progression increases TME complexity,^34,35^ single-agent therapy becomes insufficient.^36,37^ Hence, combination strategies or early interventions may be necessary for effectively targeting the IL-1β–LCN2 axis. Despite uncovering the central role of LCN2 in lung cancer BM, our study has several limitations. First, while our data strongly implicate tumor cells as the main source of functional LCN2, we cannot definitively exclude contributions from TME-derived sources (e.g., fibroblasts and granulocytes) owing to the lack of conditional LCN2 knockout models. Future studies employing cell–type–specific knockout systems are needed to fully dissect source-specific roles. Second, we did not evaluate in vivo the efficacy of LCN2-targeting therapies, such as neutralizing antibodies, which may be limited by BBB impermeability. Our results further underscore the need to develop enhanced drug delivery technologies for CNS targets. Third, the role of microglia—brain-resident macrophages—in regulating LCN2 in BM tumor cells remains to be elucidated. Fourth, the clinical efficacy of STAT3 and VEGF-A inhibition must be validated in prospective trials.

In conclusion, our findings reveal that tumor-derived LCN2 orchestrates a brain-specific metastatic program through dual mechanisms: a paracrine loop involving astrocyte activation and macrophage recruitment and a tumor-intrinsic angiogenic pathway via SLC22A17–JAK2–STAT3–VEGF-A signaling. These mechanistic insights suggest that LCN2 may serve as both a therapeutic target and a prognostic biomarker in lung cancer brain metastasis.

## Materials and methods

### Data collection

Internal data: This study included the following datasets: (1) a proprietary single-cell dataset comprising five patients diagnosed with lung cancer BM (clinical details in Supplementary Table 1); (2) formalin-fixed paraffin-embedded samples from patients with primary lung cancer and BM collected at the Cancer Hospital of the Chinese Academy of Medical Sciences up to December 2022 following surgical resection or needle biopsy of primary and/or metastatic lesions (Supplementary Table 2); (3) peripheral blood samples from treatment-naïve patients newly diagnosed with advanced lung cancer (Supplementary Table 3); (4) peripheral blood samples from healthy individuals as controls (Supplementary Table 4); and (5) previously published bulk RNA-seq data from matched primary lung tumors and corresponding BM collected at our institution.^25^ All procedures were approved by the Ethics Committee of the National Cancer Center, Cancer Hospital, Chinese Academy of Medical Sciences, Peking Union Medical College (Approval No. 23/090-3829). All participants provided informed consent for tissue and blood sample collection and single-cell RNA sequencing. The internal data are available from the corresponding author upon reasonable request.

Public data: The following public single-cell RNA-seq datasets were used: GSE202371,^38^, which contains BD Rhapsody scRNA-seq data from 10 lung cancer BM cases; GSE131907,^39^, which includes scRNA-seq profiles from 44 patients with samples spanning multiple sites; GSE234832,^40^, which contains scRNA-seq data from 3 breast cancer BM and 2 lung cancer BM cases; GSE161116,^40^, which consists of 11 matched primary lung tumors and BM from bulk RNA sequencing; and supplemental transcriptomic data from a murine pancreatic cancer model.^36^

### Generation and processing of scRNA-seq data

#### Sample preparation

Human tissue samples were processed as described above. Following tissue dissociation,^39^ single-cell RNA-seq libraries were prepared via the Chromium Single Cell 5′ Reagent Kit v2 (10x Genomics) according to the manufacturer’s protocol and sequenced on the Illumina HiSeq X-Ten platform.

#### Preprocessing and quality control

Raw FASTQ files were processed via Cell Ranger (v6.0.0) with default settings and aligned to the human reference genome (hg38). Only reads with valid barcodes and unique molecular identifiers (UMIs) that were mapped confidently were retained to generate the gene-by-cell expression matrix. Gene counts were imported into R (v4.3.1) and analyzed via the Seurat package (v4.4.0).^41^

Genes expressed in fewer than 3 cells were excluded. Potential doublets were removed via the DoubletFinder package (v2.0.4),^42^ with the doublet rate set at 0.08. Cells with fewer than 1000 UMI counts, fewer than 200 detected genes, or mitochondrial gene content exceeding 20% were discarded. The expression data were normalized via log-transformation (NormalizeData) and scaled (ScaleData). The 2000 most variable genes were identified via FindVariableFeatures (method = “vst”), and dimensionality reduction was performed via principal component analysis (RunPCA, default parameters).

To better elucidate the mechanism of LCN2 in lung cancer brain metastasis, we established two cohorts. To compare the differences between primary tumors and brain metastases, we extracted 10 BM samples and 11 primary lesion samples from GSE131907, forming Cohort 1, which consisted of 21 samples and 71,638 single cells. To compare the impact of different levels of LCN2 expression on the BM microenvironment, we integrated internal data (*n* = 5), GSE131907 (*n* = 10), GSE202371 (*n* = 10), and GSE234832 (*n* = 2) to form Cohort 2, which consisted of 27 brain metastasis samples and 108,775 single cells.

To correct batch effects and integrate datasets, we applied the Harmony algorithm. A shared nearest neighbor (SNN) graph was computed via the first 30 principal components (FindNeighbors), and clustering was performed via the matrix algorithm (FindClusters). For visualization, we used uniform manifold approximation and projection (UMAP). Cluster-specific marker genes were identified via FindAllMarkers with default parameters. Cell type annotation was based on canonical marker genes.

#### Differentially expressed gene analysis and enrichment analysis

Differentially expressed genes (DEGs) between the high- and low-LCN2 expression groups were identified via the FindMarkers function with default parameters (log2FC threshold = 0.25, adjusted *P* < 0.05). GSEA was conducted via the enrichKEGG function (ClusterProfiler V4.14.4),^43^ employing default statistical thresholds (adjusted *P* < 0.05).

#### Bulk transcriptomic data collection and analysis

We obtained bulk transcriptomic profiles of paired primary tumors and BM from two sources: GSE161116 and our previously published dataset. For GSE161116, raw count data were processed via the EdgeR package (v4.41).^44^ For our internal dataset, TPM-normalized expression data were analyzed via the limma package (v3.62.1).^45^ Differential gene expression was determined on the basis of a |log₂-fold change| > 1 and a false discovery rate (FDR) < 0.05.

#### Bulk RNA sequencing of PC9 cells

The cells were collected at approximately 80% confluence and then washed with phosphate-buffered saline. Total RNA was extracted from the cells via the TRIzol method. Sequencing and analysis were carried out by Novogene Co., Ltd. RNA integrity was validated via an Agilent 2100 bioanalyzer. Strand-specific RNA-seq libraries were assembled via the TruSeq Stranded mRNA LT Sample Prep Kit from Illumina in accordance with the manufacturer’s guidelines.

The library preparations were sequenced on an Illumina NovaSeq platform, and 150 bp paired-end reads were generated. The raw FASTQ files were processed with fastp software to remove low-quality bases and adapters. High-quality reads were aligned to the human reference genome (GRCh38) via HISAT2 (v2.0.5). Gene-level counts were generated with featureCounts, and expression was normalized as TPM. EdgeR (v3.22.5) was used to identify DEGs (adjusted *p* value < 0.05, |log2-fold change| > 1) between conditions. KEGG functional enrichment was performed with clusterProfiler.

### Experimental model and reagents

#### Cell culture

The following cell lines were used: PC9, A549, H358, H23 (all from ATCC); KLN-205 and LLC (Nanjing Kebai Biotechnology Co., Ltd); TC1 (Shanghai Binsui Biotechnology Co., Ltd); bEnd.3 (CellCook Co., Ltd); and Tohoku Hospital Pediatrics-1 (ATCC). PC9, A549, H358, H23, KLN-205, LLC, and TC1 cells were cultured in DMEM (Gibco) supplemented with 10% fetal bovine serum (FBS, Gibco) and 1% penicillin‒streptomycin (Invitrogen). bEnd.3 cells were maintained in modified DMEM (CellCook, CM2007) supplemented with 10% FBS and 1% penicillin‒streptomycin. Tohoku Hospital Pediatrics-1 cells were cultured in RPMI 1640 medium supplemented with 10% FBS and 1% penicillin‒streptomycin.

Primary mouse astrocytes were provided by the Neurology Laboratory at Tongji Medical College, HUST, and cultured in Astrocyte Medium Animal (AMA; SelenCel, cat: 35463) supplemented with 10% FBS and 1% penicillin‒streptomycin. MO3.13 human oligodendrocyte cells (Wuhan Pronosai Life Technology Co., Ltd.) were cultured in DMEM supplemented with 10% FBS and 1% penicillin‒streptomycin. All the cells were maintained in a humidified incubator at 37°C with 5% CO₂ and were routinely tested for mycoplasma contamination.

#### ShRNA sequence

human LCN2-targeting shRNA: 5′-AAGAACACCATAGCATGCTGG -3′

#### Over-expression sequence

Human LCN2 gene: atgcccctaggtctcctgtggctgggcctagccctgttgggggctctgcatgcccaggcccaggactccacctcagacctgatcccagccccacctctgagcaaggtccctctgcagcagaacttccaggacaaccaattccaggggaagtggtatgtggtaggcctggcagggaatgcaattctcaga

### RNA transfection technique

Lyophilized siRNA was reconstituted in DEPC-treated water to the desired concentration. For transfection, the siRNAs and Lipofectamine 2000 (Invitrogen) were diluted separately in serum-free and antibiotic-free DMEM. The two components were gently mixed and incubated at room temperature for 5–10 min to form RNA–lipid complexes. The cells were cultured in serum-free, antibiotic-free medium prior to transfection. The RNA–Lipofectamine complexes were added dropwise to the cells, followed by gentle swirling to ensure even distribution. After 6–8 h of incubation under standard conditions, the medium was replaced with complete DMEM containing serum and antibiotics. The cells were then cultured for 24–48 h to allow for gene knockdown or downstream analysis.

### Animal experiments

Female and male BALB/c nude and C57BL/6 J mice (4–8 weeks old) were obtained from Beijing HFK Bio-Technology Co., Ltd. and housed under specific pathogen-free conditions at the Experimental Animal Center of the Cancer Hospital, Chinese Academy of Medical Sciences (CICAMS). All procedures were conducted in accordance with protocols approved by the Animal Care and Use Committee of CICAMS (Approval Nos. NCC2020A163 and NCC2025A022).

To establish a brain metastasis (BM) model via left ventricular injection, 5 × 10⁵ luciferase-labeled PC9 (Luc-PC9) or TC1 (Luc-TC1) cells suspended in 50 μL of PBS were injected into the left cardiac ventricle via a 26 G needle in 6–8-week-old female and male BALB/c nude and C57BL/6 J mice. For the orthotopic BM model, 5 × 10⁵ cells in 5 μL of PBS were stereotactically injected into the caudate nucleus of the brain under continuous isoflurane anesthesia (RWD Life Science). Tumor growth was monitored via a Xenogen IVIS imaging system (Spectral Instrument Imaging, Lago X) coupled with Living Image Acquisition and Analysis software.

For the carotid artery injection model, 1 × 10⁵ cells suspended in 50 μL of PBS were injected directly into the carotid artery of anesthetized 6–8-week-old BALB/c nude mice (sodium pentobarbital, 1.5%, 50 mg/kg). Brain metastasis was confirmed by in vivo bioluminescence imaging (BLI) following the intraperitoneal injection of 150 μL of 1.5% D-luciferin (Beyotime Biotechnology, ST196-2G).

For the lung and liver metastasis models, 1 × 10⁶ cells in 100 μL of PBS were injected into the tail vein of 6–8-week-old BALB/c nude mice. The tumor burden was monitored weekly via BLI for up to four weeks.

For orthotopic lung tumor implantation, 2 × 10⁶ cells in 50 μL of PBS were mixed 1:1 with Matrigel (BD Biosciences) and injected into the lungs of BALB/c nude or C57BL/6 J mice. Tumor growth was evaluated weekly for four weeks via BLI.

For the subcutaneous tumor assays, 1 × 10⁶ cells in 100 μL of PBS were injected into the groin of 4–6-week-old BALB/c nude mice. The tumor volume was measured every three days via the following formula: length × width² × 0.52. The mice were euthanized via CO₂ asphyxiation, and the tumors were harvested for analysis.

At the end of each experiment or when the mice became moribund, the animals were humanely euthanized. The brains were fixed in 4% formaldehyde and processed for histopathological evaluation.

### Drug treatment

The mice were treated with bevacizumab (10 mg/kg, intraperitoneally, twice per week; MedChem Express, Cat# HY-P9906), the STAT3 inhibitor SH-4-54 (10 mg/kg, intraperitoneally, twice per week; MedChem Express, Cat# HY-16975), or the IL-1R antagonist anakinra (100 mg/kg, intraperitoneally, daily; MedChem Express, Cat# HY-108841). All drugs were dissolved in a vehicle composed of 10% dimethyl sulfoxide (DMSO; Sigma‒Aldrich, Cat# D2650), 40% PEG300 (MedChem Express, Cat# HY-Y0873), and 5% Tween-80 (MedChem Express, Cat# HY-Y1891) in saline. Vehicle-treated controls received the same formulation without active compounds. The tumor burden was assessed via BLI, and the animals were euthanized with CO₂ for tissue collection. All treatments and measurements were synchronized across groups.

### Cytokine antibody array assay

Conditioned media from PC9-BM-sh and A549-OE cells, along with their respective controls, were analyzed for cytokine and chemokine secretion via a multiplex panel that detects 45 human cytokines following the manufacturer’s protocol (Affymetrix eBioscience, EPX450-12171-901; www.eBioscience.com).

### Western blot and CoIP

Proteins were extracted from cultured cells via RIPA buffer supplemented with protease inhibitors. The samples were denatured, mixed with 1× SDS loading buffer, separated via SDS‒PAGE, and transferred to 0.2 μm PVDF membranes (Millipore). The membranes were blocked in 5% nonfat milk in TBST and incubated overnight at 4 °C with primary antibodies. After washing, HRP-conjugated secondary antibodies were applied for 1 h at room temperature. The signal was detected via enhanced chemiluminescence reagents and visualized with an ImageQuant™ LAS 4000 system.

For CoIP, lysates were incubated overnight with target antibodies at 4 °C, followed by a 30-min incubation with protein A/G beads at room temperature. The beads were washed five times with prechilled lysis buffer, resuspended in SDS loading buffer, and subjected to immunoblotting. The primary antibody RRID tags are listed in Supplemental Table 5.

### Enzyme-linked immunosorbent assay (ELISA)

The following ELISA kits were used: Lipocalin-2/NGAL (Invitrogen, EHLCN2), VEGFA (ABclonal, RK00023), and IL-1β (ABclonal, RK00001), following the manufacturers’ protocols.

### Immunofluorescence and immunohistochemistry

For immunohistochemistry (IHC), tissue sections were deparaffinized, rehydrated, and subjected to antigen retrieval with sodium citrate buffer. After being blocked with 5% goat serum for 30 min at room temperature, the sections were incubated overnight at 4°C with primary antibodies. Detection was performed using HRP-conjugated secondary antibodies (ZSGB-BIO, Cat# PV-6001 for anti-rabbit, PV-6002 for anti-mouse) and DAB chromogen. Images were captured via ImageScope (version 12.4) and analyzed with CaseViewer (3DHISTECH Ltd.), ImageJ (NIH), and Photoshop (Adobe).

For IF, tissue sections were permeabilized with PBST for 30 min and blocked with 5% goat serum in PBS for 1 h. Primary antibodies diluted in TBS-T were incubated overnight at 4 °C, followed by incubation with Alexa Fluor 488- or 594-conjugated secondary antibodies (Invitrogen, 1:1000 dilution). The slides were mounted with antifade medium containing DAPI and imaged using a ZEISS Axio Scope. A1 upright microscope. The primary RRIDs are provided in Supplemental Table 5.

### Heterogeneous cell–cell adhesion assay

To evaluate the adhesion of tumor cells to endothelial cells, bEnd.3 brain microvascular endothelial cells were seeded into 6-well plates and cultured until they reached confluence. mCherry-labeled tumor cells were then added to the endothelial monolayer and incubated for 30 min. After washing twice with PBS to remove nonadherent cells, adherent tumor cells were imaged via fluorescence microscopy and quantified per field.

### Trans-BBB migration assay

To assess transendothelial migration across the blood‒brain barrier (BBB), 5 × 10⁵ primary mouse astrocytes were seeded onto the underside of a transwell membrane. The culture medium was changed every 15 min for 6 h to support astrocyte attachment. The transwell insert was then inverted, and 2.5 × 10⁵ bEnd.3 brain endothelial cells were seeded onto the top surface. Following cell attachment, the inserts were returned to their upright position and incubated at 37 °C for 3 days to facilitate BBB formation. Afterward, transwells with intact BBB were washed twice with PBS to remove residual serum and transferred to 24-well plates containing tumor-conditioned medium collected from various tumor cell lines after a 24-hour serum starvation period. Subsequently, 4–5 × 10⁴ mCherry-labeled tumor cells were seeded into the upper chamber. Tumor cell migration across the BBB was assessed 18–22 h later.

### In vitro angiogenesis assay

An in vitro angiogenesis assay was performed using brain endothelial cells. A total of 5 × 10⁴ cells were plated onto Matrigel (Millipore In Vitro Angiogenesis Kit) in the presence of tumor-conditioned media and monitored for tube formation over 12 h. Angiogenesis was quantified by counting the number of branch points per field. The “junction points” were defined as the nodes formed at the intersections or connections between endothelial cell-derived tubular structures within a vascular network. The “branch points” were defined as the initiation sites where secondary vessels extended from a primary vessel in the vasculature.

### Cell coculture

Coculture experiments were carried out in six-well plates equipped with insert chambers (0.4 μm pore size; Corning Inc., NY, USA). THP-1 cells were pretreated with phorbol 12-myristate 13-acetate (PMA; Sigma‒Aldrich) and seeded into the lower chamber at a density of 1 × 10⁶ cells/well for 24–48 h. The tumor cells were thoroughly washed and subsequently seeded into the upper chamber. After 24 h of coculture, the tumor cells were harvested via enzymatic digestion and subjected to Western blot analysis.

### Cell migration assay

Tumor-induced macrophage migration was evaluated via the use of 24-well plates fitted with insert chambers (8 μm pores; Corning). PMA-pretreated THP-1 cells (6 × 10⁵ cells/well) were suspended in 200 μL of serum-free RPMI-1640 and seeded into the upper chamber. The lower chamber contained supernatant from cocultures of tumor cells (5 × 10⁵ cells/well) and either astrocytes or MO3.13 cells (5 × 10⁵ cells/well) and was incubated for 24 h. After an additional 24-h incubation, the migrated macrophages on the underside of the membrane were fixed with 4% paraformaldehyde for 10 min and stained with 1% crystal violet for 15 min. The number of migrated cells was quantified by counting five randomly selected fields per insert under a light microscope.

### Statistical analysis

All experiments were independently repeated at least three times unless otherwise noted. The data are presented as the means ± standard deviations. Statistical analyses were performed via GraphPad Prism (v9.5) and R software (v4.3.1). Comparisons between two groups were made via two-tailed Student’s *t*-tests. Differences were considered statistically significant at *P* < 0.05.

## Supplementary information

Supplementary-Materials

western blot

Western blot of Supplementary material

## Data Availability

The raw single-cell RNA-seq data generated in this study can be found in the Genome Sequence Archive (https://ngdc.cncb.ac.cn/gsa-human/), with the accession ID HRA013662. The BAM files for bulk RNA-seq of PC9 cells are available in the Genome Sequence Archive with the accession ID HRA014068. Publicly available scRNA-seq data were retrieved from GEO (GSE202371, GSE131907, and GSE234832).
