# Supplementary material for: Lipocalin-2 drives brain metastatic progression through reciprocal tumor-microenvironment interactions in lung cancer
Source: Signal Transduct Target Ther. 2025 Dec 24;10:417. doi: 10.1038/s41392-025-02514-2 (PMC12727695; doi:10.1038/s41392-025-02514-2)
Supplement: Supplementary file 1 — Supplementary-Materials [file 41392_2025_2514_MOESM1_ESM.docx]

Supplementary Materials for

Lipocalin-2 drives brain metastatic progression through reciprocal tumor-microenvironment interactions in lung cancer

Yixiang Zhu#, Jian Zhang#, Danming He#, Hongqing Cai, Yan He, Li Yuan, Sini Li, Yucheng Dong, Wei Zhuang, Zhijie Wang, Jianchun Duan, Xue Zhang, Zixiao Ma, Hua Bai⁎, Jie Wang⁎

Correspondence to: zlhuxi@163.com (J.W.); baihuahb@sina.com (H.B.)

**This PDF file includes:**

Figures. S1 to S10

Tables S1 to S5


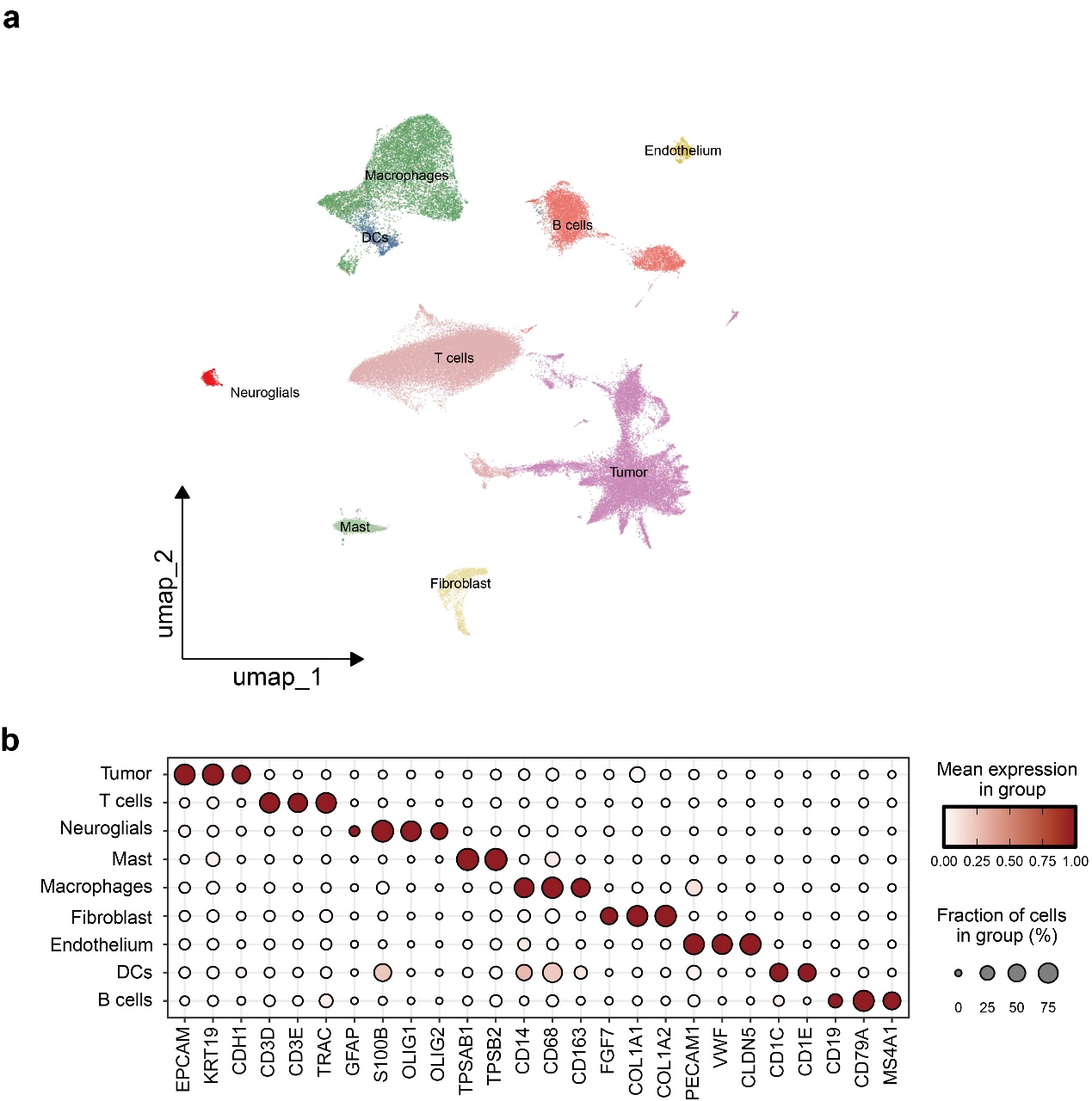


Figure. S1.

**Cell type annotation based on gene expression.** (a) Single-cell transcriptional landscape of cancer and immune cells BM of GSE131907 visualized using uniform manifold approximation and projection (UMAP). (b) Cell type annotation based on canonical gene expression profiles.


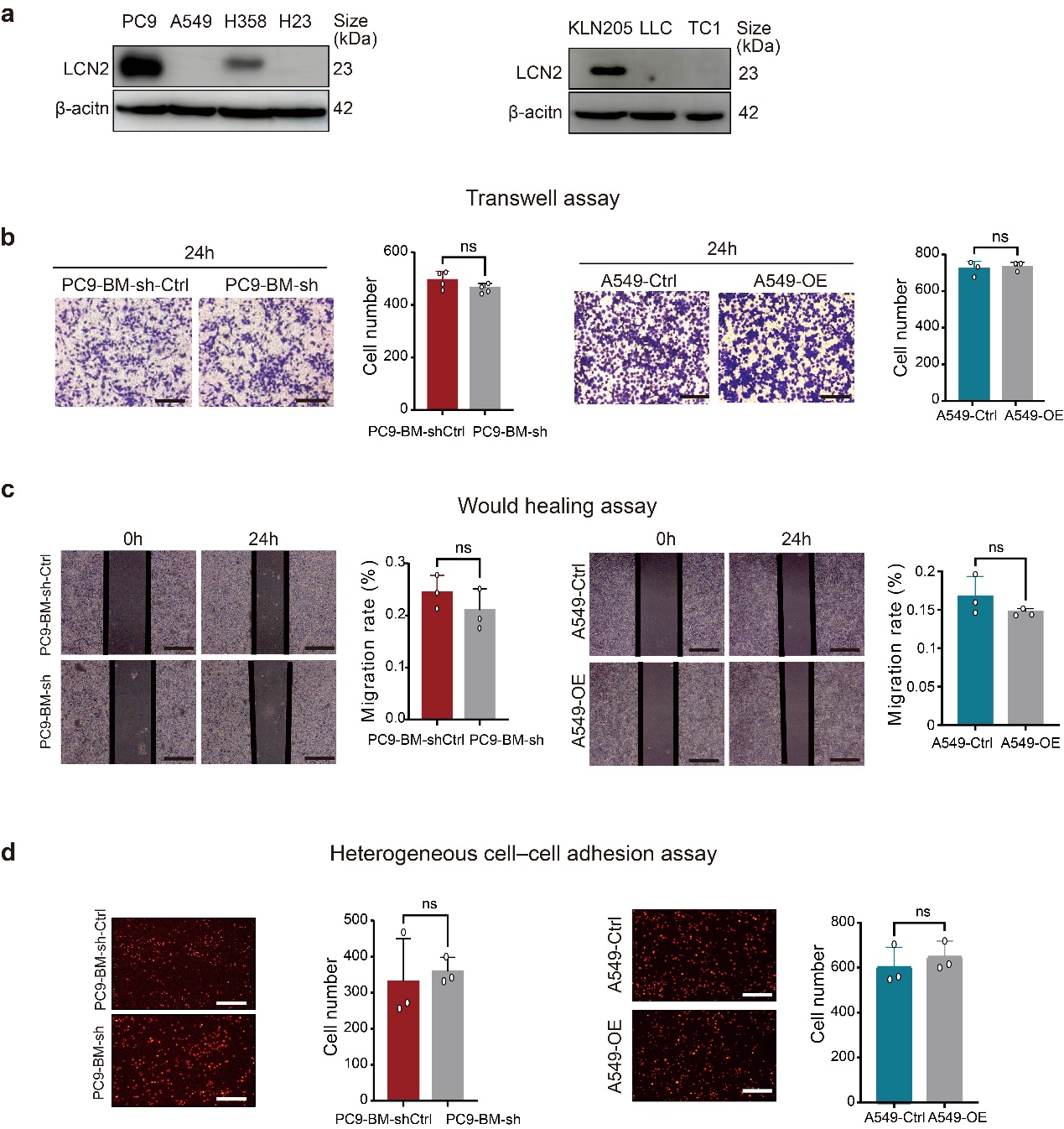


Figure. S2.

**LCN2 promotes tumor cell proliferation but does not affect migration or adhesion to brain endothelium.**  (a) Western blot analysis of LCN2 expression in PC9, A549, H358, and H23 wild-type cells. (b) Transwell migration assays of PC9-BM control (*n* = 4), PC9-BM knockdown (KD; *n* = 4), A549 control (*n* = 3), and A549 overexpression (OE; *n* = 3) cells. Data are presented as mean ± SD; two-sided *t*-test; ns, not significant. Scale bar, 200 μm. (c) Wound healing assays in PC9-BM control, PC9-BM KD, A549 control, and A549 OE cells (*n* = 3). Data are mean ± SD; two-sided *t*-test; ns, not significant. Scale bar, 200 μm. (d) Heterogeneous cell-cell adhesion assays in PC9-BM control, PC9-BM KD, A549 control, and A549 OE cells (*n* = 3). Data are mean ± SD; two-sided t-test; ns, not significant. Scale bar, 200 μm. LCN2, lipocalin-2; BM, brain metastasis; OE, overexpression.


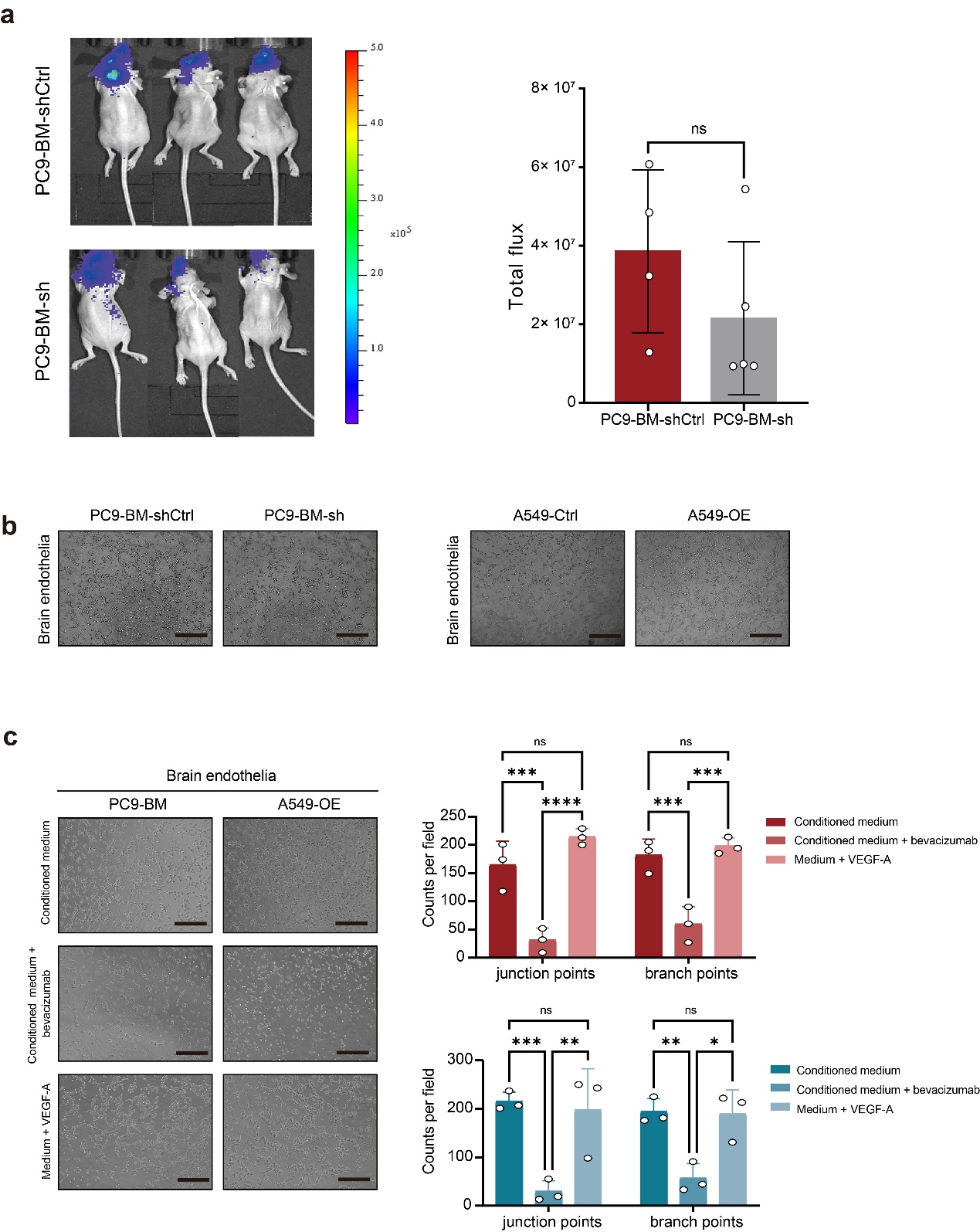


Figure. S3.

**LCN2 knockdown does not alter brain colonization by PC9-BM cells and LCN2 promotes angiogenesis in lung cancer BM.** (a) Bioluminescence imaging (BLI) 6 days after carotid artery injection of LCN2 KD (*n* = 5) and control PC9-BM cells (*n* = 4). Data are mean ± SD; two-sided t-test; ns, not significant. (b) Images of angiogenesis in brain endothelia co-cultured with medium of PC9-BM control, PC9-BM KD, A549 control, and A549 OE cells. Scale bar, 200 μm. (c) Images of angiogenesis in brain endothelia co-cultured with PC9-BM and A549 OE cells by treatment with conditioned medium, conditioned medium + bevacizumab and medium + VEGFA. Quantification of junction and branch points per field (*n* = 3) of angiogenesis in PC9-BM and A549 OE cells (*n* = 3). Data are mean ± SD; two-way ANOVA; ns, not significant. *P < 0.05; **P < 0.01; ***P < 0.001; ****P < 0.0001. Scale bar, 200 μm. BM, brain metastasis; KD, knockdown; OE, overexpression. BLI, bioluminescence imaging.


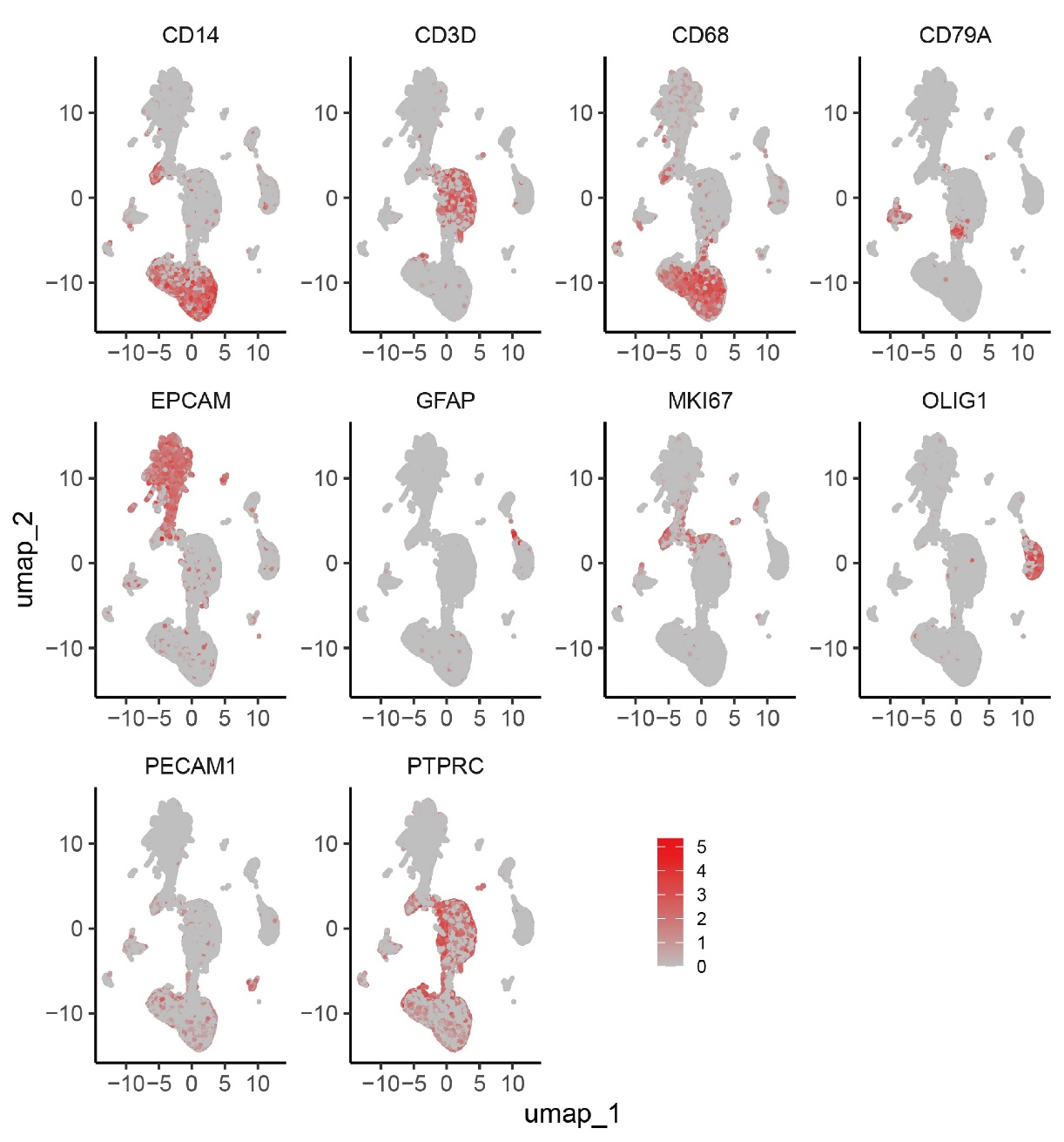


Figure. S4.

**Cell type annotation based on gene expression for 27 BM samples.**


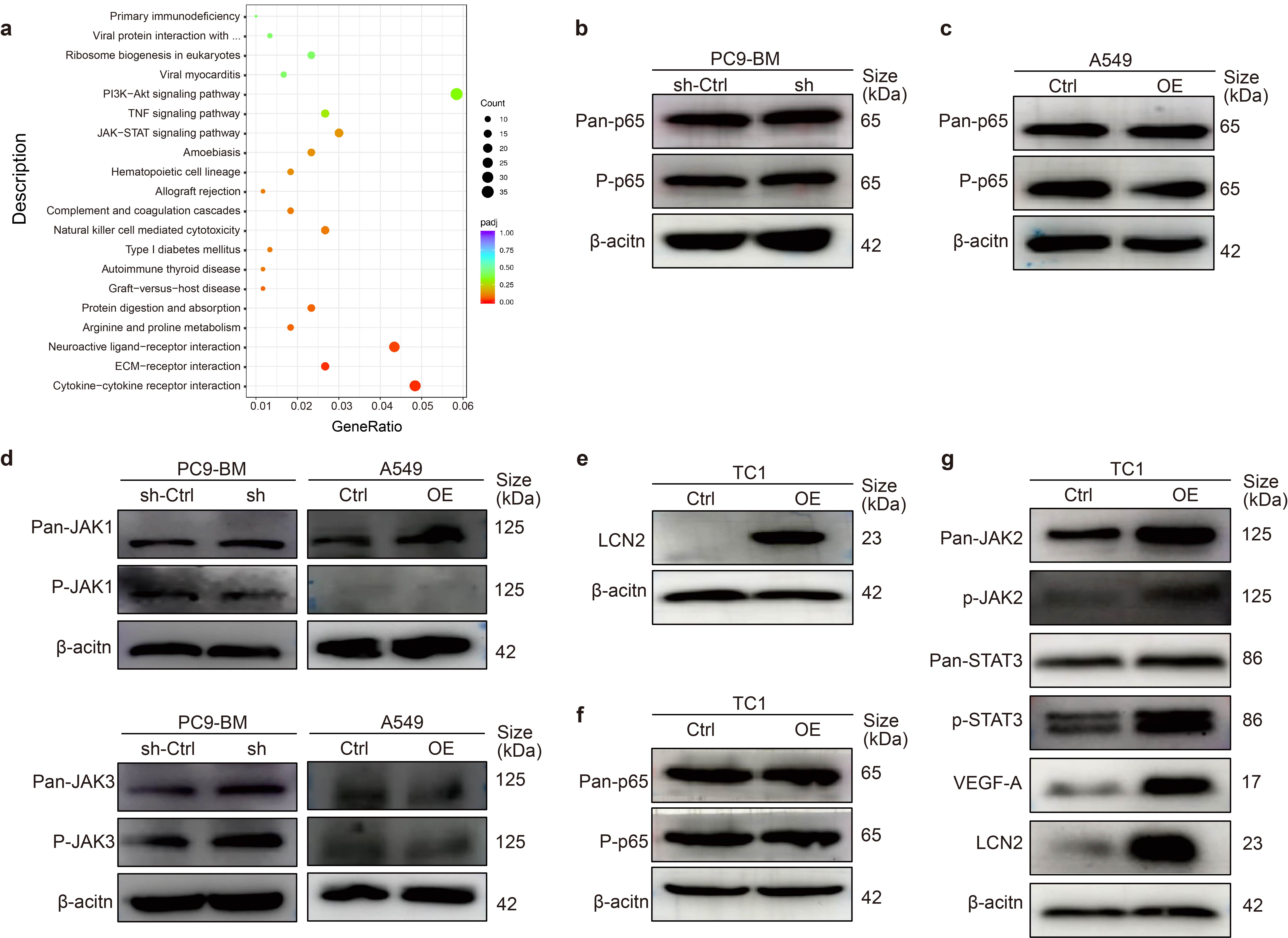


Figure. S5.

**Western blot shows p65, JAK1, and JAK3 expression.** (a) Pathway enrichment analysis of tumor cells with high versus low LCN2 expression in PC9-BM cells. (b-c) Western blot detection of p65 in PC9-BM control, PC9-BM KD, A549 control, and A549 OE cells. (d) Western blot detection of JAK1 and JAK3 in PC9-BM control, PC9-BM KD, A549 control, and A549 OE cells. (e) LCN2 expression in TC1 control and TC1 OE cells by western blot. (f) p65 expression in TC1 control and TC1 OE cells. (g) Western blot detection of phosphorylated JAK2 (p-JAK2), phosphorylated STAT3 (p-STAT3), and VEGF-A. LCN2, lipocalin-2; BM, brain metastasis; KD, knockdown; OE, overexpression.


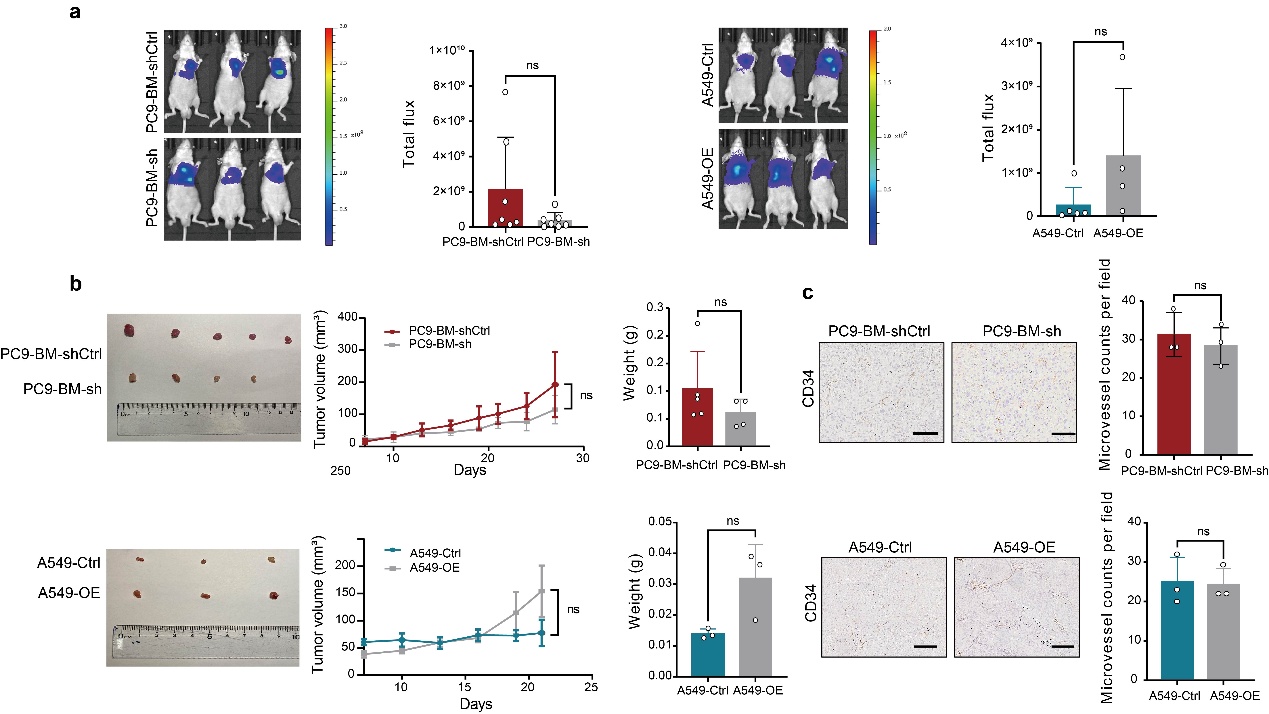


Figure. S6.

**LCN2 does not affect tumor growth in orthotopic or subcutaneous models.** (a) Tumor growth in orthotopic pulmonary transplant models using PC9-BM control (*n* = 6), PC9-BM KD (*n* = 7), A549 control (*n* = 5), and A549 OE (*n* = 4) cells. (b) Tumor growth in subcutaneous transplant models with PC9-BM control (*n* = 5), PC9-BM KD (*n* = 4), A549 control (*n* = 3), and A549 OE (*n* = 3) cells. 2 replicate experiments of A549 OE and control A549 cells. (c) Representative immunohistochemical (IHC) images for CD34 staining in subcutaneous tumors (*n* = 3). Data are mean ± SD; two-sided *t*-test; ns, not significant. Scale bar: 200 μm. LCN2, lipocalin-2; BM, brain metastasis; KD, knockdown; OE, overexpression; IHC, immunohistochemistry.


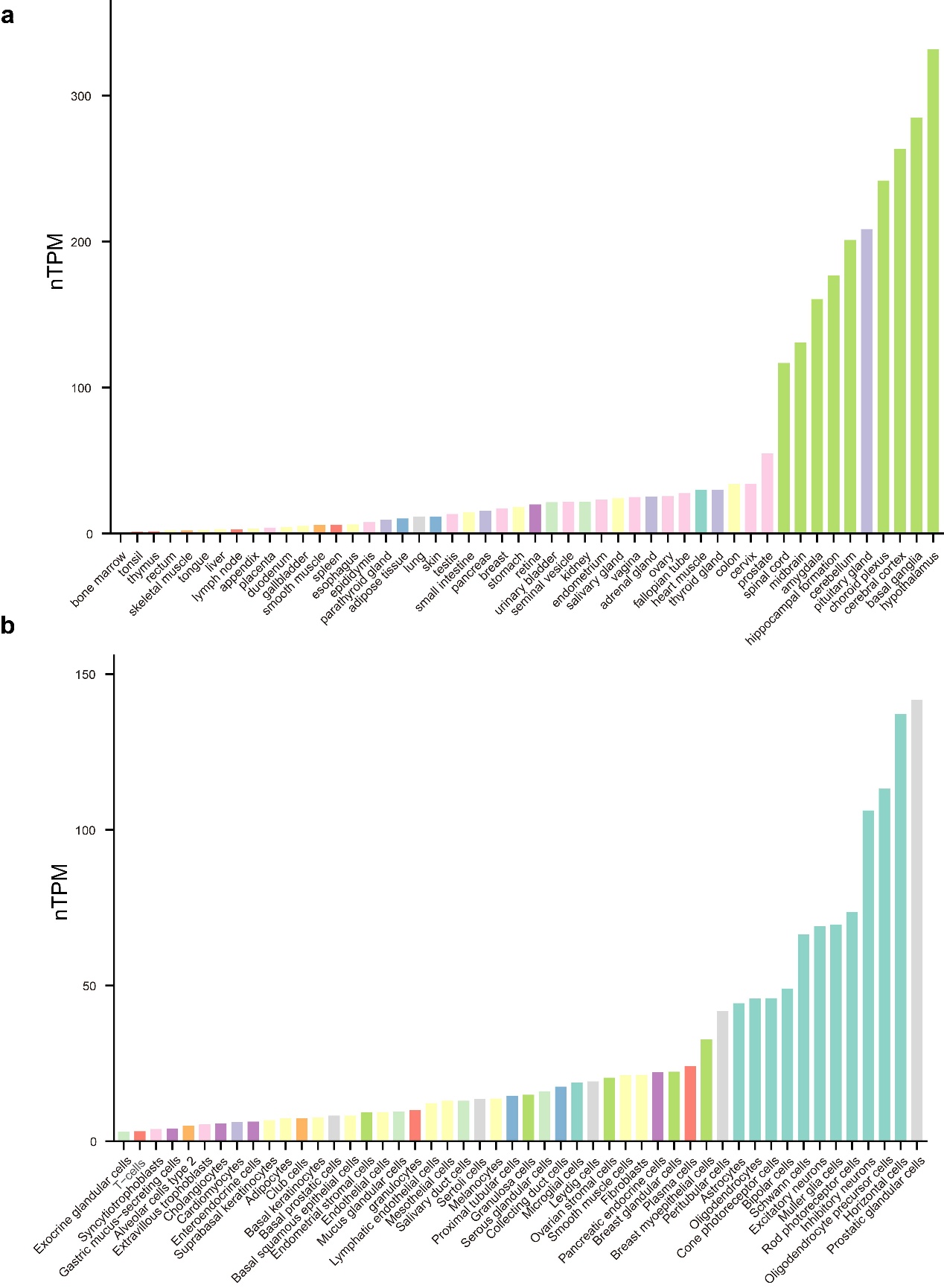


Figure. S7.

**SLC22A17 expression across tissues and cell types.** Expression profiles of SLC22A17 obtained from the Human Protein Atlas (https://www.proteinatlas.org/).


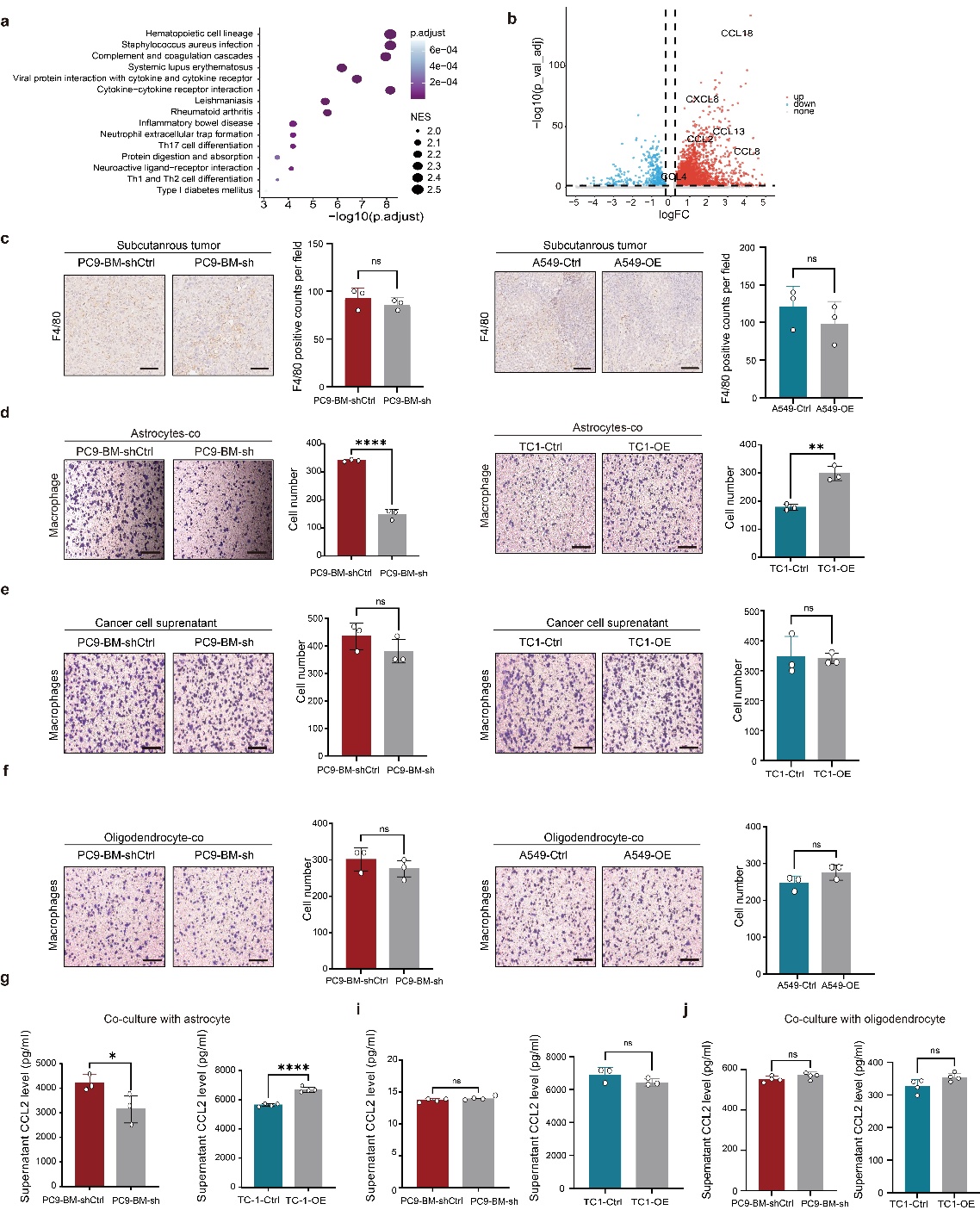


Figure. S8.

**LCN2 does not promote macrophage recruitment through tumor cells alone.** (a) Pathway enrichment in the oligodendrocyte according to the high LCN2 expression compared to the low LCN2 expression tumor cells in lung cancer patients. (b)The expression of CCL2 in the oligodendrocyte according to the high LCN2 expression compared to the low LCN2 expression tumor cells in lung cancer patients. (c) Representative IHC images of F4/80+ macrophages in subcutaneous tumor sections (*n* = 3). Data are mean ± SD; two-sided t-test, **P < 0.01, ***P < 0.001. Scale bar: 200 μm. (d) Quantification of macrophages in astrocyte–tumor cell co-cultures (n = 3); two-sided t-test, **P < 0.01, ****P < 0.0001. Scale bar, 200 μm. (e-f) Macrophage counts in media from tumor cell monocultures versus tumor cells co-cultured with oligodendrocytes (*n* = 3); two-sided t-test, ns, no statistical difference. (g) ELISA for CCL2 in astrocytes co-cultured with PC9-BM control, PC9-BM KD, TC1 control, and TC1 OE cells (*n* = 3); two-sided t-test, *P < 0.05, ****P < 0.0001. Scale bar, 200 μm. (i) ELISA for CCL2 in supernatants of tumor monocultures (*n* = 3); two-sided t-test; ns, not significant. (j) ELISA for CCL2 in oligodendrocytes co-cultured with PC9-BM control, PC9-BM KD, TC1 control, and TC1 OE cells (*n* = 4); two-sided t-test; ns, not significant. LCN2, lipocalin-2; BM, brain metastasis; OE, overexpression; IHC, immunohistochemistry.


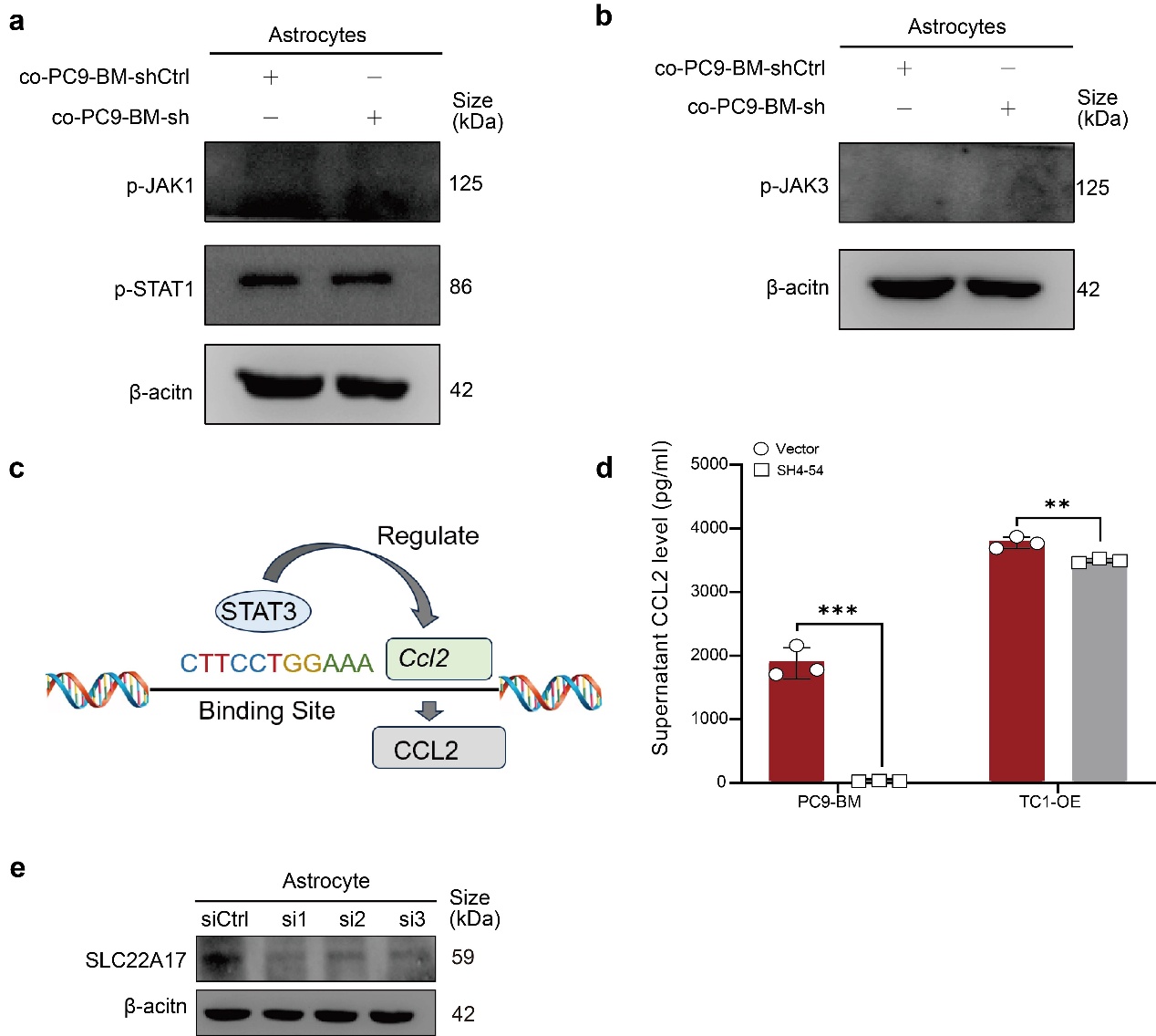


Figure. S9.

**LCN2 does not alter p-JAK1, p-STAT1, and p-JAK3 activation in astrocytes.** (a) p Western blot detection of phosphorylated JAK1 (p-JAK1) and STAT1 (p-STAT1) in astrocytes co-cultured with PC9-BM control and KD cells. (b) Detection of p-JAK3 under the same conditions. (c) Schematic illustrating STAT3 binding to the CCL2 promoter. (d) ELISA analysis of CCL2 in astrocytes co-cultured with PC9-BM control, PC9-BM KD, and TC1 OE cells, treated with STAT3 inhibitor SH4-54 (1 μM, 18 hours; *n* = 3); two-sided t-test, **P < 0.01, ***P< 0.001. (e) Western blot detection of SLC22A17 expression in astrocytes after siRNA-mediated knockdown. BM, brain metastasis; KD, knockdown.


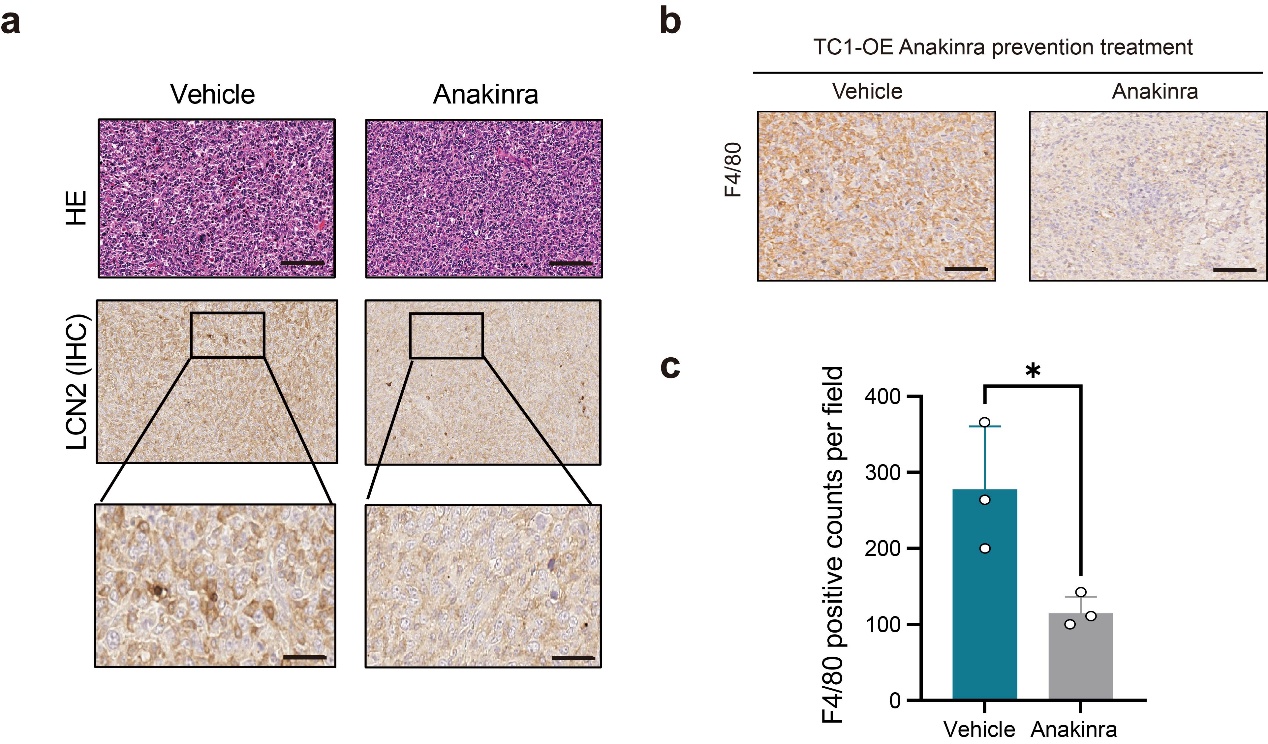


Figure. S10.

**IHC analysis of BM lesions in mice bearing TC1-OE tumors.** (a) Representative images of HE and IHC staining for LCN2 in the prevention group of mice BM. The scale bar represents 200 μm and 20 μm. (b-c) Representative images of HE and IHC staining for F4/80 in the prevention group of mice BM. Data are mean ± SD, *n* = 3; Two-sided t-test, *P< 0.05. The scale bar represents 200μm.

|  | Number (*n*) | Percentage (%) |
| --- | --- | --- |
| Sex |  |  |
| Male | 4 | 25 |
| Female | 1 | 75 |
| Age |  |  |
| ≥65 years old | 2 | 40 |
| ＜65 years old | 3 | 60 |
| Pathology |  |  |
| Adenocarcinoma | 5 | 100 |

Table S1. Clinical baseline characteristics of 5 patients with sc-RNA.

|  | Number (*n*) | Percentage (%) |
| --- | --- | --- |
| Sex |  |  |
| Male | 39 | 53.4 |
| Female | 34 | 46.6 |
| Age |  |  |
| ≥65 years old | 20 | 27.4 |
| ＜65 years old | 53 | 72.6 |
| Pathology |  |  |
| Adenocarcinoma | 56 | 76.7 |
| Squamous cell carcinoma | 9 | 12.3 |
| Adenosquamous carcinoma | 1 | 1.3 |
| Small cell | 4 | 5.5 |
| Large cell neuroendocrine | 2 | 2.7 |
| Unknown | 1 | 1.3 |
| BM type |  |  |
| Synchroneity | 20 | 27.4 |
| Metachronous | 53 | 72.6 |
| BM LCN2 score |  |  |
| 3+ | 21 | 28.8 |
| ＜3+ | 52 | 71.2 |
| Primary tissue LCN2 score |  |  |
| 3+ | 7 | 16.3 |
| ＜3+ | 36 | 83.7 |

LCN2, Lipocalin-2; IHC, Immunohistochemistry.

Table S2. Clinical baseline characteristics of 73 BM patients with IHC.

|  | Number (*n*) | Percentage (%) |
| --- | --- | --- |
| Pathology |  |  |
| Adenocarcinoma | 24 | 61.5 |
| Squamous cell carcinoma | 2 | 5.1 |
| Small cell | 10 | 25.7 |
| Other | 3 | 7.7 |
| Metastasis site |  |  |
| Brian | 25 | 27.8 |
| Liver | 14 | 14.4 |

LCN2, Lipocalin-2.

Table S3. Clinical baseline characteristics of 39 patients with Elisa testing.

|  | Number (*n*) | Percentage (%) |
| --- | --- | --- |
| Sex |  |  |
| Male | 3 | 30 |
| Female | 7 | 70 |
| Age |  |  |
| ≥65 years old | 0 | 0 |
| ＜65 years old | 10 | 100 |

Table S4. Baseline characteristics of 10 healthy patients with Elisa testing.

|  | Brand | Cat No. | Reaction species | Usage |
| --- | --- | --- | --- | --- |
| CD34 | Abcam | ab81289 | Rabbit | 1:2000 (IHC) |
| IBA-1 | ABclonal | A19776 | Rabbit | 1:300 (IHC) |
| F4/80 | Cell Signaling | 70076 | Mouse | 1:300 (IHC) |
| CD68 | ZSGB-Bio | ZM-0060 | - | 1:1 (IHC) |
| LCN2 | Cell Signaling | 44058 | Rabbit | 1:300 (IHC/IF) |
| LCN2 | Santa | Sc-518095 | Mouse | 1:300 (IHC/IF) |
| SLC22A17 | Sigma-Aldrich | SAB3500306 | Rabbit | 1:300 (IF) |
| p-JAK2 | Cell Signaling | 3771S | Rabbit | 1:300 (IF) |
| VEGF-A | Cell Signaling | 50661S | Rabbit | 1:1000 (Western blot) |
| STAT3 | Cell Signaling | 9139 | Mouse | 1:1000 (Western blot) |
| p-STAT3 | Cell Signaling | 9145 | Rabbit | 1:1000 (Western blot) |
| JAK3 | Cell Signaling | 8827 | Rabbit | 1:1000 (Western blot) |
| p-JAK3 | Cell Signaling | 5031 | Rabbit | 1:1000 (Western blot) |
| JAK2 | Cell Signaling | 3230 | Rabbit | 1:1000 (Western blot) |
| p-JAK2 | Cell Signaling | 3771S | Rabbit | 1:1000 (Western blot) |
| JAK1 | Cell Signaling | 50996 | Mouse | 1:1000 (Western blot) |
| p-JAK1 | Cell Signaling | 74129 | Rabbit | 1:1000 (Western blot) |
| SLC22A17 | Sigma-Aldrich | SAB3500306 | Rabbit | 1:500 (Western blot) |
| β-actin | ZSGB-Bio | TA-09 | Mouse | 1:1000 (Western blot) |
| Anti-mouse IgG | Cell Signaling | 7074 | Mouse | 1:3000 (Western blot) |
| Anti-rabbit IgG | Cell Signaling | 70767 | Rabbit | 1:3000 (Western blot) |
| Lipocalin-2/NGAL Protein | MCE | HY-P70658A | Mouse | 100 ng/mL |
| Lipocalin-2/NGAL Protein | MCE | HY-P71156 | Human | 100 ng/mL |
| Anti-mouse LCN2 antibody | R&D | MAB1857 | Mouse | 100 ng/mL |
| IL-1 beta Protein | MCE | HY-P7028 | Human | 100 ng/ml |
| IL-1 beta Protein | MCE | HY-P7073 | Mouse | 100 ng/ml |

IHC, Immunohistochemistry, IF, immunofluorescence

Table S5. Antibodies used for immunohistochemistry, immunofluorescence, and protein.
