# Supplementary figures and images for: Lipocalin-2 drives brain metastatic progression through reciprocal tumor-microenvironment interactions in lung cancer
Source: Signal Transduct Target Ther. 2025 Dec 24;10:417. doi: 10.1038/s41392-025-02514-2 (PMC12727695; doi:10.1038/s41392-025-02514-2)

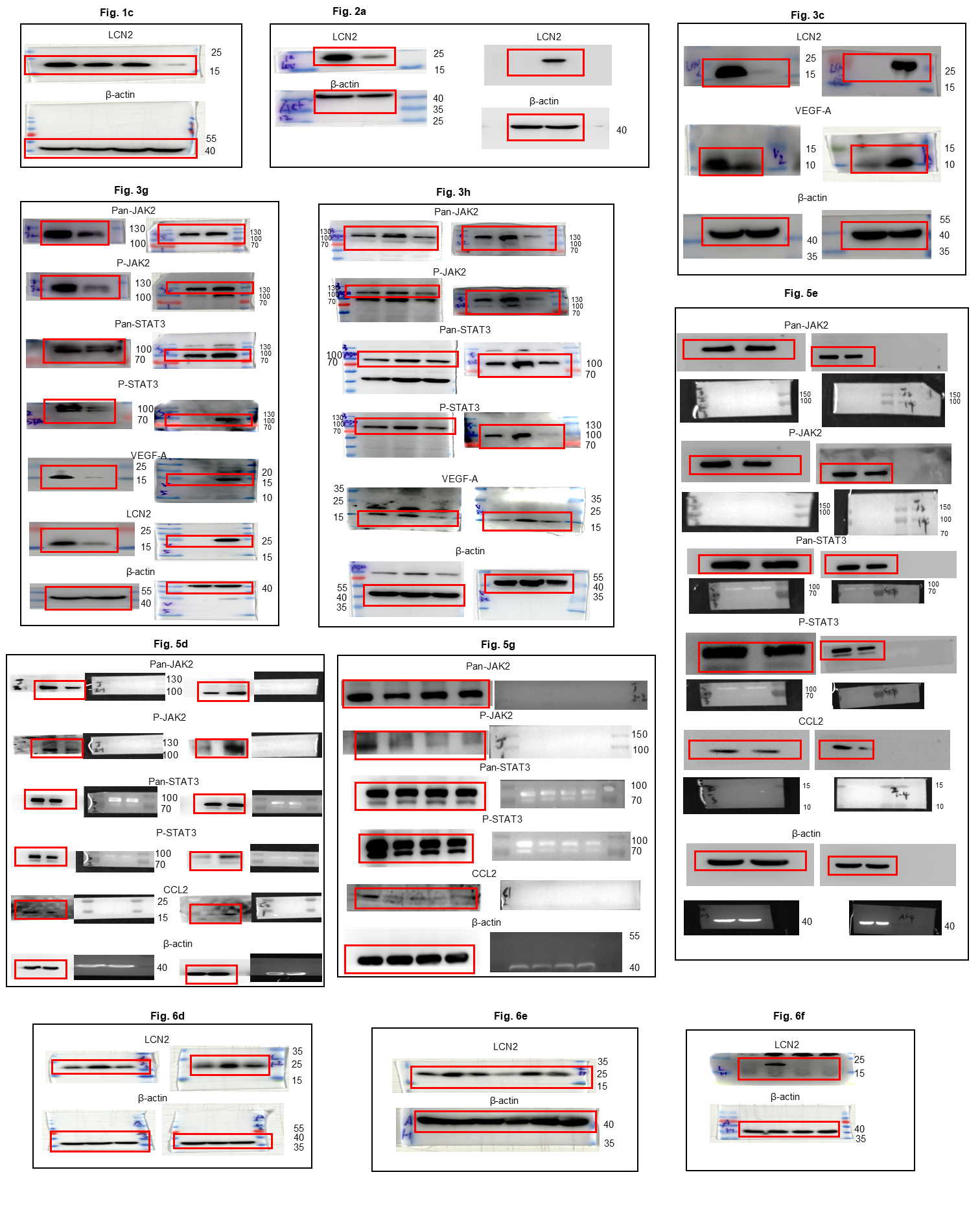

Supplement: Supplementary file 2 — western blot [file 41392_2025_2514_MOESM2_ESM.png]

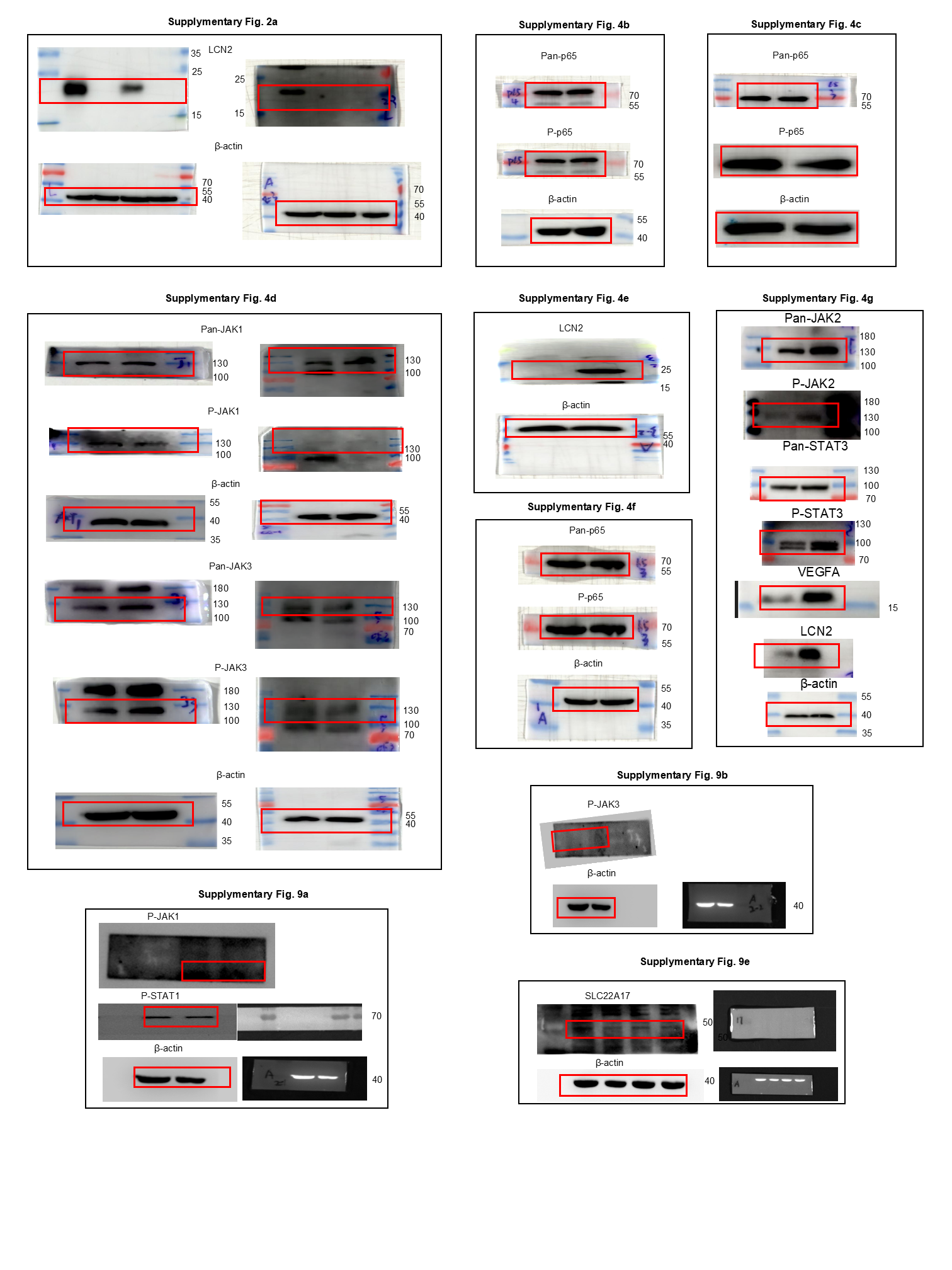

Supplement: Supplementary file 3 — Western blot of Supplementary material [file 41392_2025_2514_MOESM3_ESM.png]
